# The hydroperoxyl antiradical activity of natural hydroxycinnamic acid derivatives in physiological environments: the effects of pH values on rate constants†

**DOI:** 10.1039/d2ra02311c

**Published:** 2022-05-18

**Authors:** Nguyen Thi Hoa, Le Thi Ngoc Van, Quan V. Vo

**Affiliations:** The University of Danang – University of Technology and Education Danang 550000 Vietnam vvquan@ute.udn.vn vovanquan1980@gmail.com; Duy Tan University Da Nang 550000 Vietnam

## Abstract

Hydroxycinnamic acid derivatives (HCA) are a type of phenolic acid that occurs naturally. HCA are widely known for their anti-inflammatory, anti-cancer, and especially antioxidant capabilities; however, a comprehensive study of the mechanism and kinetics of the antiradical activity of these compounds has not been performed. Here, we report a study on the mechanisms and kinetics of hydroperoxyl radical scavenging activity of HCA by density functional theory (DFT) calculations. The ability of HCA to scavenge hydroperoxyl radicals in physiological environments was studied. The results showed that HCA had moderate and weak HOO˙ antiradical activity in pentyl ethanoate solvent, with the overall rate constant *k*_overall_ = 8.60 × 10^1^ − 3.40 × 10^4^ M^−1^ s^−1^. The formal hydrogen transfer mechanism of phenyl hydroxyl groups defined this action. However, in water at physiological pH, 2-coumaric acid (1), 4-coumaric acid (2), caffeic acid (3), ferulic acid (4), sinapic acid (5) and 4-hydroxyphenylpyruvic acid (7) exhibit a significant HOO˙ antiradical activity with *k*_total_ = 10^5^ − 10^8^ M^−1^ s^−1^ by the electron transfer reaction of the phenolate anions. Following a rise in pH levels in most of the studied substances, the overall rate constant varied. The acid 5 exhibited the highest HOO˙ radical scavenging activity (log(*k*_overall_) = 4.6–5.1) at pH < 5; however, at pH = 5.4–8.8, the highest HOO˙ radical scavenging activity were observed for 3 with log(*k*_overall_) = 5.2–5.7. At pH > 6.2, acids 2, 3, 4, and 5 presented the largest radical scavenging activity. By contrast, acid 3-coumaric acid (8) had the lowest antiradical activity at most pH values. Thus, the hydroperoxyl radical scavenging activity in pentyl ethanoate follows the order 3 > 5 > 1 ∼ 2 ∼ 4 ∼ 6 (homovanillic acid) ∼ 7 > 8, whereas it follows the order 3 > 2 ∼ 4 ∼ 5 > 6 ∼ 7 > 1 > 8 in water at pH = 7.40. The activity of 1, 2, 3, 4, 5, 6, and 7 are faster than those of the reference Trolox, suggesting that these HCA could be useful natural antioxidants in the aqueous physiological environment.

## Abstract 2

Quantum chemistry calculations suggest that hydroxycinnamic acid derivatives are good natural antioxidants in aqueous solutions.

## Graphical abstract

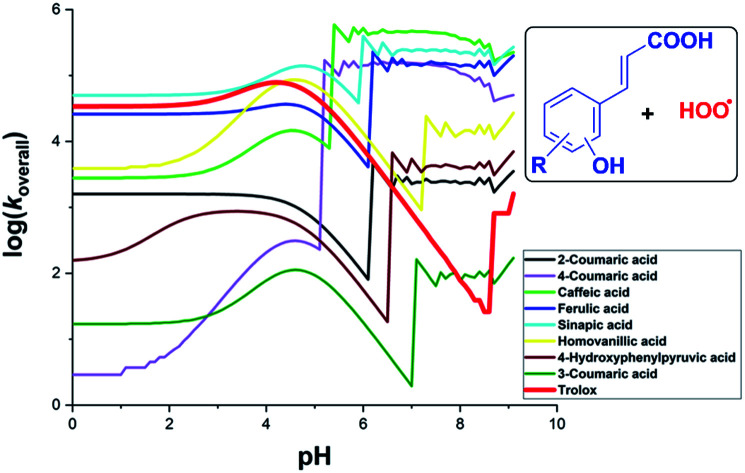

## 1. Introduction

**Fig. 1:**
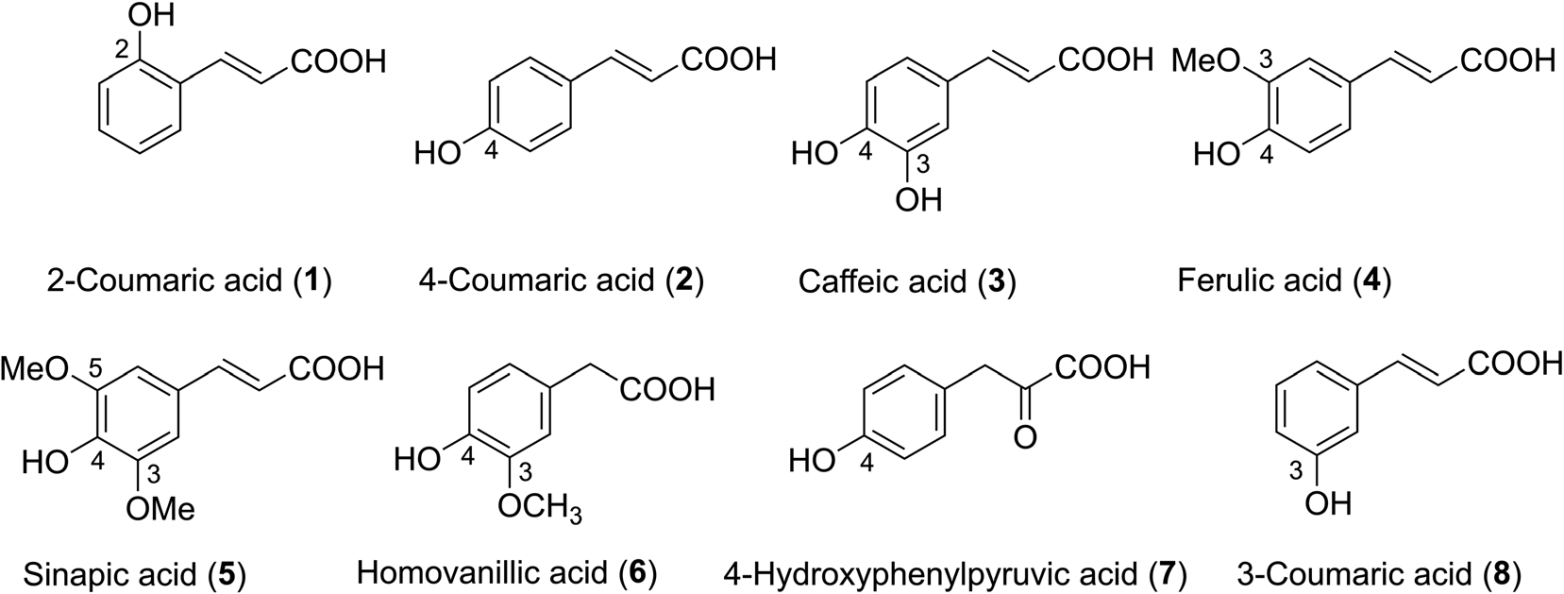
Structure of hydroxycinnamic acid derivatives (HCA).

Phenolic acids are found in practically all plant-based foods and make up a large part of the human diet. The typical daily consumption of phenolic acid in humans has been estimated to be around 200 mg, depending on food patterns and preferences.^1^ Hydroxycinnamic acid derivatives (HCA, Fig. 1) are a type of phenolic acid that occurs naturally. They are secondary plant metabolites generated from phenylalanine and tyrosine, and they all have a C6C3 carbon skeleton with a cis or trans double bond in the side chain. Among the most well-known HCA are 2-coumaric acid (1), 3-coumaric acid (8), 4-coumaric acid (2), caffeic acid (3), ferulic acid (4), and sinapic acid (5) (Fig. 1).^2^ Homovanillic acid (6) is a key catecholamine metabolite formed when monoamine oxidase and catechol-*O*-methyltransferase operate on dopamine in a sequential manner,^3^ however 4-hydroxyphenylpyruvic acid (7) is an intermediate in the tyrosine aminotransferase's metabolism of the amino acid phenylalanine *via* tyrosine.^4^HCA has attracted much attention because they are the most important antioxidants in our diet.^5–10^ Nenadis *et al.* reported HCA*i.e.*3, 4 and 5 exhibited a good radical scavenging activity *via* the 2,2-diphenyl-1-picrylhydrazyl (DPPH˙) and 2,2′-azinobis(3-ethylbenzothiazoline-6-sulfonic acid) (ABTS˙^+^) assays.^11^ Kadoma and co-workers reported that acids 2 or 3/2-mercaptoethanol mixtures might have a synergistic or antagonistic effect *in vivo*, implying their powerful chemopreventive efficacy against chronic illnesses and cancers.^12^HCA showed considerable radical scavenging action in computational studies, which backed up the experimental findings.^13–15^ The studies showed that 2 and 3 exhibited potent hydroxyl radical scavenging activity,^14,15^ whereas 1 presented good antiradical activity against HOO˙, CH_3_O˙ and CH_3_OO˙.^14^ The study on 3 and its derivatives indicated that the inhibitors of peroxidation of HCA increased in the rise in pH levels and the activity could be better than that of Trolox at pH = 8.^16^ It is deserved to have a broader investigation of the effects of pH values on the mechanism and especially the kinetics of the antiradical activity of this family compounds; however, this is yet to be performed. Thus in this study, the hydroperoxyl radical scavenging activity of typical hydroxycinnamic acids in physiological environments was investigated by thermodynamic and kinetic calculations. The effect of pH on the activity was also investigated.

## 2. Computational details

All computations in this work were performed using the Gaussian 09 suite of programs,^17^ which used the density functional theory (DFT) method. The M06-2X functional and the 6-311++G(d,p) basis set were used to carry out all of the calculations.^18^ The M06-2X functional is one of the most reliable approaches for studying radical reaction thermodynamics and kinetics,^19,20^ with only minor inaccuracies when compared to experimental data (*k*_calc_/*k*_exp_ ratio = 1–2.9),^21–25^ and is widely used to assess the radical scavenging activity of natural and synthetic compounds.^36–39^ The solvation model based on density (SMD) method^26^ was used to predict the solvent effects of water and pentyl ethanoate, which is commonly used for modelling the radical scavenging activity of antioxidants.^19,25,27^ The quantum mechanics-based test for overall free radical scavenging activity (QM-ORSA) methodology was used to accomplish the kinetic calculations.^24^ The rate constant (*k*) was calculated by using the conventional transition state theory (TST) (at 298.15 K, 1 M standard state) according to the eqn (1):^25,28–34^1
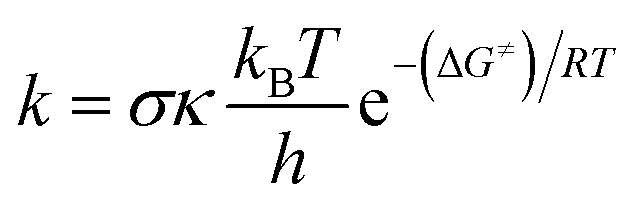
where: *σ* is the reaction symmetry number,^35,36^*κ* contains the tunneling corrections calculated using the Eckart barrier,^37^*k*_B_ is the Boltzmann constant, *h* is the Planck constant, Δ*G*^≠^ is the Gibbs free energy of activation.

The Marcus theory was used to estimate the reaction barriers of single electron transfer (SET) reactions.^38–41^ The free energy of reaction Δ*G*^≠^ for the SET pathway was computed following the eqn (2) and (3).2
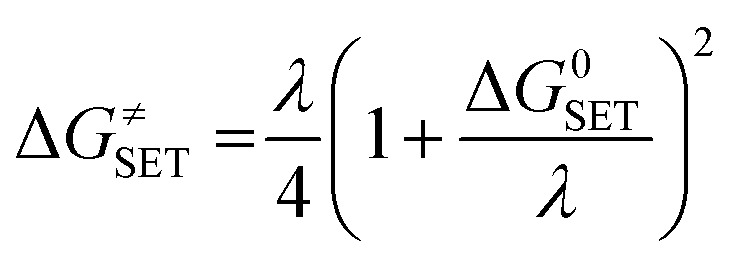

3 *λ* ≈ Δ*E*_SET_ − Δ*G*^0^_SET_

where Δ*G*_SET_ is the Gibbs energy of reaction, Δ*E*_SET_ is the non-adiabatic energy difference between reactants and vertical products for SET.^42,43^

For rate constants that were close to the diffusion limit, a correction was applied to yield realistic results.^24^ The apparent rate constants (*k*_app_) were calculated following the Collins–Kimball theory in the solvents at 298.15 K;^44^ the steady-state Smoluchowski rate constant (*k*_D_) for an irreversible bimolecular diffusion-controlled reaction was calculated following the literature as corroding to eqn (4) and (5).^24,45^4
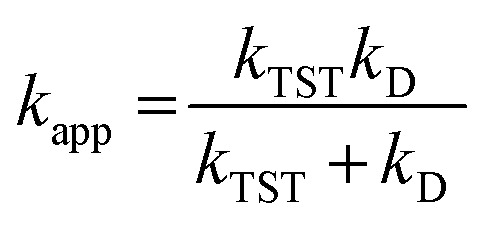

5 *k*_D_ = 4π*R*_AB_*D*_AB_*N*_A_

where *R*_AB_ is the reaction distance, *N*_A_ is the Avogadro constant, and *D*_AB_ = *D*_A_ + *D*_B_ (*D*_AB_ is the mutual diffusion coefficient of the reactants A and B),^44,46^ where *D*_A_ or *D*_B_ is estimated using the Stokes–Einstein formulation (6).^47,48^6
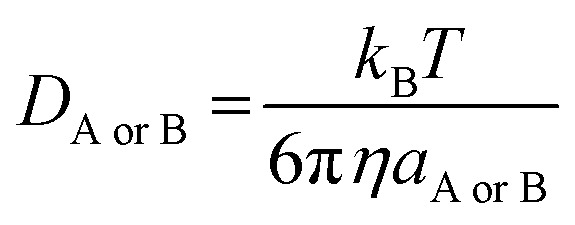


*η* is the viscosity of the solvents (*i.e. η*(H_2_O) = 8.91 × 10^−4^ Pa s, *η*(pentyl ethanoate) = 8.62 × 10^−4^ Pa s) and *a* is the radius of the solute. More details on the method can be found in Table S1, ESI.†

## 3. Results and discussion

### 3.1 The HOO radical scavenging activity of HCA in the lipid medium

Previous research has demonstrated that HOO˙ radical addition reactions do not occur at either C=C bonds or the aromatic ring system,^23,27,49–52^ thus, this mechanism was not examined in this study. It was also found that the formal hydrogen transfer (FHT) mechanism, which is defined by BDE values, is the primary antiradical pathway in nonpolar environments.^53–55^ The activity of HCA was initially tested in lipid media (*i.e.* pentyl ethanoate) to see if it could scavenge radicals. As a result, the BDE values for all of the essential O–H bonds were computed in the lipid medium and are shown in Table 1. The BDEs were found to have a range of 80.1 to 98.3 kcal mol^−1^. The lowest BDE was observed at the O4–H bond at compound 3, whereas that of 8 was the highest. The *o*-hydroxy derivative (1) has the lowest BDE value (85.4 kcal mol^−1^) when substituent positions (*o*, *m*, and *p*) are considered. The *p*-hydroxycinnamic acid (2, BDE(O4–H) = 86.7 kcal mol^−1^) has a small rise in BDE, while the *m*-hydroxycinnamic acid (8) has the largest at 98.3 kcal mol^−1^. In comparison with 2–O4–H, the presence of hydroxyls and/or methoxy groups at the 3 and/or 5 positions (compounds 3, 4, and 5) could reduce BDE(O4–H) by roughly 2.4–6.6 kcal mol^−1^. In the presence of hydroxyl groups, however, the decrease of BDE(O4–H) was larger (compound 3) than in the presence of methoxy groups (compounds 4 and 5).

**Table 1:** The BDE of the O–H bond, Δ*G*^0^, Δ*G*^≠^ (in kcal mol^−1^), rate constants (*k*_app_, and *k*_overall_ M^−1^ s^−1^) of the HCA + HOO˙ reactions in pentyl ethanoate

Comp.	Positions	BDE	Δ*G*^0^	Δ*G*^≠^	*k*_app_	*k*_Trolox_/*k*_HCA_^a^
1	O2–H	85.4	−1.1	16.8	6.30 × 10^2^	158.7
2	O4–H	86.7	0.9	18.6	4.00 × 10^2^	250.0
3	O3–H	82.1	−4.7	14.5	2.30 × 10^4^	
	O4–H	80.1	−6.8	14.7	1.10 × 10^4^	
	*k*_overall_				**3.40** × **10**^**4**^	2.9
4	O4–H	84.3	−1.7	18.4	1.80 × 10^2^	555.6
5	O4–H	81.9	−4.3	15.9	9.00 × 10^3^	11.1
6	O4–H	85.2	−0.8	17.1	6.40 × 10^2^	156.3
7	O4–H	87.9	1.6	18.1	5.70 × 10^2^	175.4
8	O3–H	98.3	3.6	19.2	8.60 × 10^1^	1162.8

a *k*_Trolox_ (calculated) = 1.00 × 10^5^ (ref. 56).

Kinetic calculations were performed to assess the HOO˙ radical scavenging capacity of HCA. The rate constants of the HCA + HOO˙ reactions in the nonpolar environment range between 8.60 × 10^1^ to 3.40 × 10^4^ M^−1^ s^−1^ (Table 1). Based on the kinetic data, the HOO˙ antiradical activity of HCA in pentyl ethanoate follows the order 3 > 5 > 1, 2, 4, 6, 7 > 8. Compared with a typical antioxidant (Trolox),^56^ the HOO˙ antiradical activity of HCA in the lipid medium is lower than that of Trolox (by about 3–1000 times). Thus HCA are moderate and weak radical scavengers in the lipid medium.

### 3.2 The HOO radical scavenging activity of HCA in water

#### 3.2.1. Acid–base equilibrium

Deprotonation has been shown to have a critical role in the radical scavenging action of phenolic acids in water in previous investigations.^55,57–59^ The deprotonation equilibria and molar fraction (*f*) of each compound were examined in the first step, and the findings are reported in Table 2. The p*K*_a_ values of 1, 2, 3, 4, 5, 6, and 8 were taken from the previous studies,^60–63^ whereas that of 7 was computed following the literature,^64^ which is widely used to calculate p*K*_a_ values of carboxylic and phenol groups with good accuracy (mean unsigned errors < 0.35 p*K*_a_ units, deviations from experiments < 0.5 p*K*_a_ units),^65–67^ due to a lack of experimental data. It was found that most of the acids can be deprotonated in two steps with the *f* values (in 1 mol) range of 0.000–0.003, 0.902–0.998 and 0.001–0.097 for H_2_A (neutral), HA (anion), A (dianion) states, respectively, accepted for 3 which can be deprotonated in three steps and this acid exists mostly as the anion (*f* = 0.937) and dianion (*f* = 0.062) states in pH = 7.40. Thus, in the aqueous physiological environment, all of the states were used to evaluate the radical scavenging activity of HCA.

**Table 2:** The p*K*_a_ and molar fraction (*f*) values of HCA at pH = 7.40

Comp.	p*K*_a_ (group)			*f* (in 1 mol)			
	p*K*_a_1__	p*K*_a_2__	p*K*_a_3__	H_3_A	H_2_A	HA	A
1^a^	4.13(COOH)	9.48 (O2–H)			0.001	0.991	0.008
2^b^	4.39 (COOH)	8.37 (O4–H)			0.001	0.902	0.097
3^c^	4.38 (COOH)	8.58 (O4–H)	11.50 (O3–H)	0.001	0.937	0.062	0.000
4^d^	4.56 (COOH)	9.39 (O4–H)			0.001	0.988	0.011
5^d^	4.90 (COOH)	9.20 (O4–H)			0.003	0.981	0.016
6^d^	4.41 (COOH)	10.52 (O4–H)			0.001	0.998	0.001
7^e^	2.04 (COOH)	9.81 (O4–H)			0.000	0.996	0.004
8^a^	4.48 (COOH)	10.35 (O3–H)			0.001	0.998	0.001

a Ref. 60.

b Ref. 61.

c Ref. 62.

d Ref. 63.

e Calculated in this work.

#### 3.2.2. Thermodynamic evaluation

The antiradical scavenging activity in water can be mediated by competing for FHT and SET reactions in all the states, including neutral, anion, dianion, and trianion. As a result, the BDE(OH) of these states were calculated in water and presented in Table 3. The BDE in the neutral state (HCA) varies from 82.9 to 91.7 kcal mol^−1^. The 8–O2–H bond had the highest BDE, whereas the 3–O4–H bond had the lowest in all of the HCA. The computed BDEs in the lipid environment agree well with this. The impact of the existence and position of OH and MeO substituents on the aromatic ring (*o*, *m*, *p*) on the BDEs(O–H) showed comparable tendencies to those observed in the lipid medium. In comparison to the *p* and *o*-hydroxyl derivatives (1 and 2), the *m*-hydroxyl derivative (8) has the greatest BDE value (91.7 kcal mol^−1^), and the existence of hydroxyl or methoxy groups at the 3 and/or 5 positions (3, 4 and 5) can diminish BDE(O4–H). The formation of intramolecular hydrogen bonds^68–70^ and the delocalization of unpaired electrons over the aromatic ring due to the presence of the electron-donating groups *i.e.* HO and MeO^68,71^ can explain this decrease. The anion or dianion states (HCA-ANION, HCA-DIANION) have lower BDE(O–H) than the comparable neutral states. The greatest and smallest BDEs, however, are found at the 8–O3–H and 3–O4–H bonds, respectively. The findings suggest that the H-abstraction of anion or dianion states may be easier than that of neutral states.

**Table 3:** The calculated BDE(O–H) and Δ*G*^0^ (kcal mol^−1^) of the HCA + HOO˙ reactions according to the FHT mechanism in water

Comp	Positions	HCA		HCA-ANION		HCA-DIANION	
		BDE	Δ*G*^0^	BDE	Δ*G*^0^	BDE	Δ*G*^0^
1	O2–H	87.7	−1.9	87.4	−2.3		
2	O4–H	89.2	0.5	86.7	−1.6		
3	O3–H	84.7	−3.4	83.3	−5.3	74.7	−14.2
	O4–H	82.9	−5.4	80.9	−7.9		
4	O4–H	84.2	−5.4	82.9	−8.0		
5	O4–H	83.1	−5.8	81.2	−7.3		
6	O4–H	84.6	−4.5	82.8	−6.4		
7	O4–H	89.7	0.5	88.6	−0.5		
8	O3–H	91.7	2.3	90.7	1.9		

#### 3.2.3. The kinetics of the reactions of HCA with HOO˙ in water at the physiological pH

**Fig. 2:**
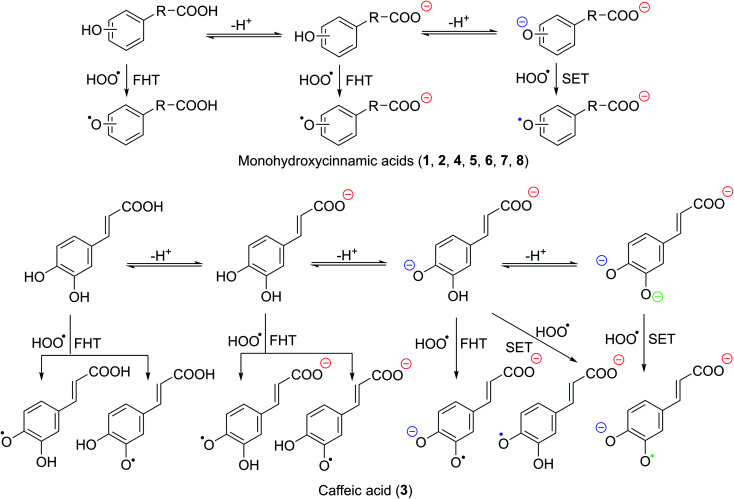
The selected mechanisms of the HOO˙ + HCA reactions in water.

The kinetics of HCA + HOO˙ reactions in water were estimated using the competing FHT and SET processes, as in prior research.^55,65,67,72^Eqn (7) was used to obtain the rate constants of the states (*k*_state_), while eqn (8)–(10) were used to calculate the rate constant incorporating the molar fraction (*k*_f_), the total rate constant (*k*_total_) and the overall rate constant (*k*_overall_) that included the *f* value of HOO˙ (p*K*_a_(HOO˙) = 4.8).^73^ Table S2, ESI,† and Table 4 describe the findings, whereas Fig. 2 depicts chosen mechanisms.

7 *k*_state_ = *k*_app_(SET) + ∑*k*_app_(FHT)

8 *k*_f_ = *f* × *k*_state_

9 *k*_total_ = ∑*k*_f_

10 *k*_overall_ = *f*(HOO˙) × *k*_total_

**Table 4:** Calculated *k*_state_, and, *k*_f_, *k*_total_, *k*_overall_ (M^−1^ s^−1^) and branching ratios (*Γ*, %) at 298.15 K, in the reactions of the HCA with HOO˙ in the aqueous solution^a^

Comp.		*k*_state_	*f*	*k*_f_	*Γ*	Comp.		*k*_state_	*f*	*k*_f_	*Γ* b
1	H_2_A	1.60 × 10^3^	0.001	1.6	0.0	5	H_2_A	5.00 × 10^4^	0.003	1.50 × 10^2^	0.0
	HA^−^	1.70 × 10^3^	0.991	1.68 × 10^3^	0.2		HA^−^	5.70 × 10^5^	0.981	5.59 × 10^5^	0.6
	A^2−^	1.20 × 10^8^	0.008	9.60 × 10^5^	99.8		A^2−^	6.10 × 10^9^	0.016	9.76 × 10^7^	99.4
	***k***_**total**_			**9.62** × **10**^**5**^			***k***_**total**_			**9.82** × **10**^**7**^	
	***k***_**overall**_			**2.41** × **10**^**3**^			***k***_**overall**_			**2.46** × **10**^**5**^	
2	H_2_A	5.90 × 10^2^	0.001	0.59	0.0	6	H_2_A	3.90 × 10^3^	0.001	3.9	0.0
	HA^−^	8.20 × 10^2^	0.902	7.40 × 10^2^	0.0		HA^−^	2.30 × 10^5^	0.998	2.30 × 10^5^	3.0
	A^2−^	6.00 × 10^8^	0.097	5.82 × 10^7^	100.0		A^2−^	7.30 × 10^9^	0.001	7.30 × 10^6^	97.0
	***k***_**total**_			**5.82** × **10**^**7**^			***k***_**total**_			**7.53** × **10**^**6**^	
	***k***_**overall**_			**1.46** × **10**^**5**^			***k***_**overall**_			**1.88** × **10**^**4**^	
3	H_3_A	2.79 × 10^3^	0.001	2.79	0.0	7	H_2_A	1.50 × 10^2^	0.000	0	0.0
	H_2_A^−^	3.65 × 10^4^	0.937	3.42 × 10^4^	0.0		HA^−^	9.40 × 10^2^	0.996	9.36 × 10^2^	0.1
	HA^2−^	2.90 × 10^9^	0.062	1.80 × 10^8^	100.0		A^2−^	4.30 × 10^8^	0.004	1.72 × 10^6^	99.9
	A^3−^	7.90 × 10^9^	0.000	0	0.0		***k***_**total**_			**1.72** × **10**^**6**^	
							***k***_**overall**_			**4.30** × **10**^**3**^	
	***k***_**total**_			**1.80** × **10**^**8**^		8	H_2_A	1.70 × 10^1^	0.001	1.70 × 10^−2^	0.0
	***k***_**overall**_			**4.50** × **10**^**5**^							
4	H_2_A	2.60 × 10^4^	0.001	2.60 × 10^1^	0.0		HA^−^	3.10 × 10^2^	0.998	3.09 × 10^2^	1.0
	HA^−^	8.80 × 10^4^	0.988	8.69 × 10^4^	0.1		A^2−^	3.20 × 10^7^	0.001	3.20 × 10^4^	99.0
	A^2−^	5.90 × 10^9^	0.011	6.49 × 10^7^	99.9		***k***_**total**_			**3.23** × **10**^**4**^	
	***k***_**total**_			**6.50** × **10**^**7**^			***k***_**overall**_			**8.08** × **10**^**1**^	
	***k***_**overall**_			**1.63** × **10**^**5**^							

a *k*_total_ (Trolox, calculated) = 1.3 × 10^5^ M^−1^ s^−1^,^56^*k*_overall_ (Trolox, calculated) = 3.25 × 10^2^ M^−1^ s^−1^.^74^

b *Γ* = *k*_f_ × 100/*k*_total_.

As shown in Table 4, all of the studied acids exhibit a significant HOO˙ scavenging activity with *k*_total_ = 3.23 × 10^4^ − 1.80 × 10^8^ M^−1^ s^−1^ and *k*_overall_ = 8.08 × 10^1^ − 4.50 × 10^5^ M^−1^ s^−1^. The activity is defined by the dianion states (*Γ* = 97.0–100%). By contrast, the neutral and anion states have no contributions (*Γ* < 3%) whatsoever in the overall rate constant of the HCA + HOO˙ reactions in water at pH = 7.40. The HOO˙ radical scavenging activity of HCA was likewise discovered to be defined by the SET pathway of the phenolate anions, with the FHT reaction playing a minimal role. That is why the antiradical activity of HCA in water at physiological pH is substantially faster than in nonpolar solvents. The total rate constant was unaffected by the SET reaction of carboxyl anion states (COO^−^, Table S2, ESI†). As a result, when evaluating the HOO˙ antiradical activity of phenolic acids in water, this reaction should be skipped (Fig. 2); however, the SET reaction of phenolate anion must be considered. The antiradical activity of 3 is the highest, with *k*_total_ = 1.80 × 10^8^ M^−1^ s^−1^. Acids 2, 4, and 5 have the second highest activity (*k*_total_ = ∼10^7^ M^−1^ s^−1^), which is more than ten times higher than acids 6 and 7. The lowest rate constant was observed at 8 with *k*_total_ = 3.23 × 10^4^ M^−1^ s^−1^. That is good in line with the result at the lipid medium.

Thus, based on the computed data, the hydroperoxyl radical scavenging activity of HCA in water at pH = 7.40 follows the order 3 > 2 ∼ 4 ∼ 5 > 6 ∼ 7 > 1 > 8. Compared with Trolox (*k*_total_ = 1.30 × 10^5^ M^−1^ s^−1^,^56^ or 8.96 × 10^4^ M^−1^ s^−1^ (M05-2X)^22^) the kinetics of 1, 2, 3, 4, 5, 6, and 7 are faster. Therefore, these HCA are promising natural antioxidants in the aqueous physiological environment.

#### 3.2.4. The effect of pH value on the reactions of HCA with HOO˙ in water

**Fig. 3:**
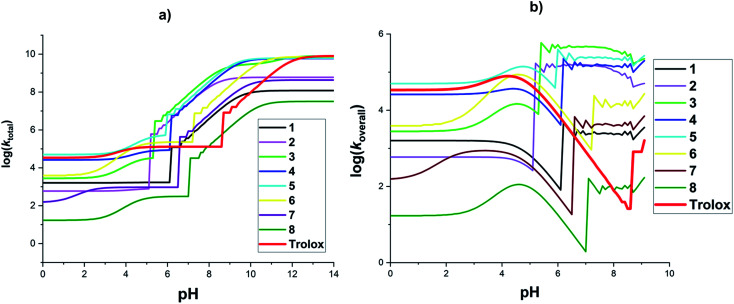
Calculated log(*k*_total_) (a) and log(*k*_overall_) (b) at 298.15 K, in the reactions of the HCA with HOO˙ in water following pH values.

The effects of pH values on the rate constant of the reactions was also explored. Fig. 3, Tables S3 and S4 ESI† show the final results. For the total rate constant (Fig. 3a), the log(*k*_total_) increased following the rise in pH values. The log(*k*_total_) rose slightly at pH < 5 and then grew significantly by about 4–6 units in the range of pH = 5–10 and reached the highest point and remained constant beyond pH = 12. The rapid increase in the log(*k*_total_) values at pH ∼ 5–10, due to the much higher p*K*_a_2__(H_2_A) or p*K*_a_3__(H_3_A) values compared with p*K*_a_1__(2) (Table 2) and the high speed SET reactions of the phenolate anion states. However, the HCA + HOO˙ reactions occurred slowly under acidic conditions (pH < 4). That is because, in these pH levels, most of the studied compounds exist at the neutral or monoanion (COO^−^) states and their SET and FHT reactions were slow.

It is noticed that the *f* value of HOO˙ is zero at pH > 9.1 due to p*K*_a_(HOO˙) = 4.80. Thus the effects of pH values on the overall rate constant of the HCA + HOO˙ reaction were only investigated at pH ≤ 9.1 (Fig. 3b). It was found that the overall rate constant varied following the rise in pH values. There was an increase in the log(*k*_overall_) all most of the studied acids at pH < 4.5, after slightly declining, the overall rate constants rose significantly at pH = 5–9 and then fluctuated. The overall rate constant were zero at pH > 9.2 due to the *f*(HOO˙) = 0 at this pH level. The acid 5 exhibited the highest HOO˙ antiradical activity (log(*k*_overall_) = 4.6–5.1) at pH < 5; however, at pH = 5.4–8.8, the highest HOO˙ radical scavenging activities were observed for 3 with log(*k*_overall_) = 5.2–5.7. At pH > 6.2, acids 2, 3, 4, and 5 presented the highest radical scavenging activity. By contrast, the HOO˙ radical trapping of 8 was lowest in all of the pH levels. At pH 5–8, the log(*k*_overall_) values increased rapidly due to the substantially greater p*K*_a_2__(H_2_A) or p*K*_a_3__(H_3_A) values relative to p*K*_a_1__(2) (Table 2). Compared with a reference compound (Trolox),^74^ at pH < 4, most of the studied acids (apart from compound 5) exhibited lower antiradical activity than Trolox, whereas at pH > 6.6 all of HCA exhibited higher HOO˙ radical scavenging activity than Trolox (apart from 8) because of the significant increase in rate constants of HCA + HOO˙ reactions at pH = ∼5–9.

## 4. Conclusion

The hydroperoxyl radical scavenging capacity of HCA in physiological media was investigated. The results showed that HCA exhibited moderate and low HOO˙ antiradical activity in the nonpolar environment with the *k*_overall_ = 8.60 × 10^1^ to 3.40 × 10^4^ M^−1^ s^−1^. This activity was defined by the FHT mechanism of hydroxyl groups. However, in water at physiological pH, the acids 1, 2, 3, 4, 5, and 7 exhibit a significant HOO˙ antiradical activity with *k*_total_ = 10^5^ − 10^8^ M^−1^ s^−1^ and *k*_overall_ = 8.08 × 10^1^ − 4.50 × 10^5^ M^−1^ s^−1^ by the SET reaction of the phenolate anions. It was found that the *k*_total_ of HCA + HOO˙ reactions increased, but the *k*_overall_ varied following the rise in pH values. The acid 5 exhibited the highest HOO˙ antiradical activity (log(*k*_overall_) = 4.6–5.1) at pH < 5; however, at pH = 5.4–8.8, the highest HOO˙ radical scavenging activities were observed for 3 with log(*k*_overall_) = 5.2–5.7. At pH > 6.2, acids 3, 4, and 5 presented the highest radical scavenging activity. By contrast, acid 8 had the lowest antiradical activity at all of the pH values. Thus based on the computed data, the hydroperoxyl radical scavenging activity in pentyl ethanoate follows the order 3 > 5 > 1, 2, 4, 6, 7 > 8, whereas that at water at pH = 7.40 follows the order 3 > 2 ∼ 4 ∼ 5 > 6 ∼ 7 > 1 > 8. The activities of 1, 2, 3, 4, 5, 6, and 7 are faster than that of the reference compound Trolox, and thus these HCA are promising natural antioxidants in the aqueous physiological environment.

## Data availability

All relevant necessary data to reproduce all results in the paper are within the main text and ESI file.† The Cartesian coordinates, the frequency, and energies of transition states for running calculations are also included in the ESI file.†

## Conflicts of interest

There are no conflicts to declare.

## Supplementary Material

RA-012-D2RA02311C-s001
