# Supplementary material for: The hydroperoxyl antiradical activity of natural hydroxycinnamic acid derivatives in physiological environments: the effects of pH values on rate constants
Source: RSC Adv. 2022 May 18;12(24):15115–22. doi: 10.1039/d2ra02311c (PMC9115882; doi:10.1039/d2ra02311c)
Supplement: RA-012-D2RA02311C-s001 [file RA-012-D2RA02311C-s001.pdf]

Electronic Supplementary Material (ESI) for RSC Advances.
This journal is © The Royal Society of Chemistry 2022

Supporting Information (SI)

The hydroperoxyl antiradical activity of natural hydroxycinnamic acid derivatives in physiological environments: The effects of pH values on rate constants

Nguyen Thi Hoa[1], Le Thi Ngoc Van[2] and Quan V. Vo[1*]

[1]The University of Danang - University of Technology and Education, Danang 550000, Vietnam.

[2]Duy Tan University, Da Nang 550000, Vietnam

**Corresponding authors: vvquan@ute.udn.vn;*

Table of Contents

Figure S1. The optimized structures of TS of the HOO$^{\bullet}$ + HCA reactions following the FHT mechanism in pentyl ethanoate S2

Table S1. The method to calculate rate constant following the conventional transition state theory S3

Table S2. Calculated $\Delta G^{\neq}$ (kcal/mol), rate constants (k_{app}, and, k_{state}, k_f, k_{total}, $k_{overall}$ $M^{-1} s^{-1}$), and branching ratios (Γ, %) at 298.15 K, in the reactions of the HCA with HOO$^{\bullet}$ in the aqueous solution at pH = 7.40........ S5

Table S3: Calculated log(k_{total}) at 298.15 K, in the reactions of the HCA with HOO• in water following pH values S7

Table S4: Calculated log($k_{overall}$) at 298.15 K, in the reactions of the HCA with HOO• in water following pH values S13

Table S5: The Cartesian coordinates and energies of TS of the reaction between HCA with HOO$^{\bullet}$ following the FHT mechanism in studied solvents........ S17

References S31

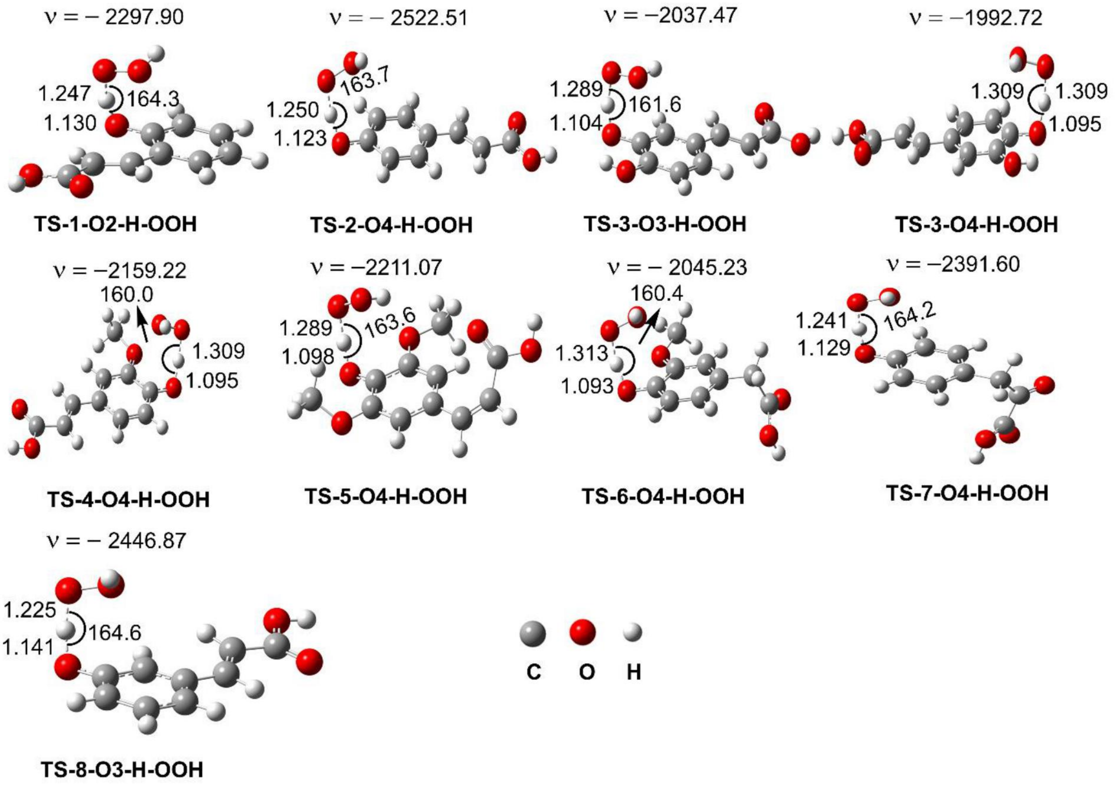

Figure S1. The optimized structures of TS of the HOO• + HCA reactions following the FHT mechanism in pentyl ethanoate

Table S1. The method to calculate rate constant following the conventional transition state theory

The rate constant (k) was calculated by using the conventional transition state theory (TST) (at 298.15 K, 1M standard state) according to the equation (1):[1-5]
$$k = \sigma\kappa \frac{k_B T}{h} e^{-(\Delta G^{\neq})/RT} \quad (1)$$
Where: σ is the reaction symmetry number ,[6,7]
κ contains the tunneling corrections calculated using the Eckart barrier,[8]
k_B is the Boltzmann constant,
h is the Planck constant,
$\Delta G^{\neq}$ is the Gibbs free energy of activation.
The Marcus Theory was used to estimate the reaction barriers of SET reactions.[9-12] The free energy of reaction $\Delta G^{\neq}$ for the SET pathway was computed following the equations (2,3).
$$\Delta G^{\neq}_{SET} = \frac{\lambda}{4}\left(1 + \frac{\Delta G^0_{SET}}{\lambda}\right)^2 \quad (2)$$
$$\lambda \approx \Delta E_{SET} - \Delta G^0_{SET} \quad (3)$$
where ΔG_{SET} is the Gibbs energy of reaction, ΔE_{SET} is the non-adiabatic energy difference between reactants and vertical products for SET.[13,14]
For rate constants that were close to the diffusion limit a correction was applied to yield realistic results[15]. The apparent rate constants (k_{app}) were calculated following the Collins–Kimball theory in the solvents at 298.15K;[16] the steady-state Smoluchowski rate constant (k_D) for an irreversible bimolecular diffusion–controlled reaction was calculated following the literature as corroding to equations (4,5).[15,17]
$$k_{app} = \frac{k_{TST} k_D}{k_{TST} + k_D} \quad (4)$$
$$k_D = 4\pi R_{AB} D_{AB} N_A \quad (5)$$

where R_{AB} is the reaction distance, N_A is the Avogadro constant, and $D_{AB} = D_A + D_B$ (D_{AB} is the mutual diffusion coefficient of the reactants A and B),[16,18] where D_A or D_B is estimated using the Stokes–Einstein formulation (6).[19,20]

$$D_{A\ or\ B} = \frac{k_B T}{6\pi \eta a_{A\ or\ B}} \quad (6)$$

η is the viscosity of the solvents (i.e. $\eta(H_2O) = 8.91\times10^{-4}$ Pa s, η(pentyl ethanoate) $= 8.62\times10^{-4}$ Pa s) and *a* is the radius of the solute.

The kinetic study requires different considerations. In this study, the solvent effects of water and pentyl ethanoate were modelled by the solvation model density (SMD) method [21,22]. Water (dielectric constants, $\varepsilon = 78.35$) and pentyl ethanoate ($\varepsilon = 4.73$) are the *de facto* standard solvents in the literature to mimic the polar and nonpolar environments in the human body [15,23-25]. Thus, these solvents were used to model the physiological environments. The solvent cage effects were included following the corrections proposed by Okuno,[26] adjusted with the free volume theory according to the Benson correction[15,27-29] to reduce over-penalizing entropy losses in solution. For the species that have multiple conformers, all of these were investigated and the conformer with the lowest electronic energy was included in the analysis.[24,25] The hindered internal rotation treatment was also applied to the single bonds to ensure that the obtained conformer has the lowest electronic energy [25,30]. All transition states were characterized by the existence of only one single imaginary frequency. Intrinsic coordinate calculations (IRCs) were performed to ensure that each transition state is connected correctly with the pre-complex and post-complex.

Table S2. Calculated $\Delta G^{\neq}$ (kcal/mol), rate constants (k_{app}, and, k_{state}, k_f, k_{total}, $k_{overall}$ M^{-1} s^{-1}), and branching ratios (Γ, %) at 298.15 K, in the reactions of the HCA with $HOO^{\bullet}$ in the aqueous solution at pH = 7.40.

Comp.	States	Mechanisms		$\Delta G^{\neq}$	κ	k_{app}	k_{state}	f	k_f	Γ
1	**H2A**	SET		44.2	17.9	2.40E-20	1.60E+03	0.001	1.60E+00	0
		FHT	O2-H	18	3749.6	1.60E+03				
	**HA-**	SET		37.7	18	1.50E-15	1.70E+03	0.991	1.68E+03	0.2
		FHT	O2-H	17.7	2647.4	1.70E+03				
	**A2-**	SET		6.4	15.1	1.20E+08	1.20E+08	0.008	9.60E+05	99.8
	**k_{total}**								**9.62E+05**	
	**$k_{overall}$**								**2.40E+03**	
2	**H2A**	SET		40.1	16.4	2.70E-17	5.90E+02	0.001	0.59	0
		FHT	O4-H	20.0	45083.3	5.90E+02				
	**HA-**	SET		30.3	17.2	4.10E-10	8.20E+02	0.902	7.40E+02	0
		FHT	O4-H	18.9	9681.2	8.20E+02				
	**A2-**	SET		5.4	14.6	6.00E+08	6.00E+08	0.097	5.82E+07	100
	**k_{total}**								**5.82E+07**	
	**$k_{overall}$**								**1.46E+05**	
3	**H3A**	SET		34.8	16.3	1.80E-13	2.79E+03	0.001	2.79E+00	0
		FHT	O3-H	16.3	261.6	1.80E+03				
			O4-H	17.1	582.4	9.90E+02				
	**H2A-**	SET		26.5	17.6	2.40E-07	3.65E+04	0.937	3.42E+04	0
		FHT	O3-H	15.6	353.1	8.50E+03				
			O4-H	14.4	152.6	2.80E+04				
	**HA2-**	SET		4.3	15.7	2.90E+09	2.90E+09	0.062	1.80E+08	100
		FHT	O3-H	12.1	1	8.00E+03				
	**A3-**	SET		0.1	16.5	7.90E+09	7.90E+09	0	-8.70E-07	0
	**k_{total}**								**1.80E+08**	
	**$k_{overall}$**								**4.50E+05**	
4	**H2A**	SET		30.3	17.8	3.80E-10	2.60E+04	0.001	2.60E+01	0
		FHT	O4-H	16.2	2938.2	2.60E+04				

	**HA-**	SET		24.4	18.1	8.40E-06	8.80E+04	0.988	8.69E+04	0.1
		FHT	O4-H	13.9	229.9	8.80E+04				
	**A2-**	SET		3.3	15.6	5.90E+09	5.90E+09	0.011	6.49E+07	99.9
	k_{total}								**6.50E+07**	
	$k_{overall}$								**1.62E+05**	
5	**H2A**	SET		32.1	18	1.80E-11	5.00E+04	0.003	1.50E+02	0
		FHT	O4-H	15.1	936	5.00E+04				
	**HA-**	SET		24.9	18.7	3.70E-06	5.70E+05	0.981	5.59E+05	0.6
		FHT	O4-H	14	1696.7	5.70E+05				
	**A2-**	SET		3.2	15.9	6.10E+09	6.10E+09	0.016	9.76E+07	99.4
	k_{total}								**9.82E+07**	
	$k_{overall}$								**2.45E+05**	
6	**H2A**	SET		29.3	18.9	2.10E-09	3.90E+03	0.001	3.90E+00	0
		FHT	O4-H	15.8	252	3.90E+03				
	**HA-**	SET		24.8	18.7	4.30E-06	2.30E+05	0.998	2.30E+05	3
		FHT	O4-H	14.5	1475.8	2.30E+05				
	**A2-**	SET		2.4	17.5	7.30E+09	7.30E+09	0.001	7.30E+06	97
	k_{total}								**7.53E+06**	
	$k_{overall}$								**1.88E+04**	
7	**H2A**	SET		40.7	18	8.60E-18	1.50E+02	0	0.00E+00	0
		FHT	O4-H	19.8	7995.4	1.50E+02				
	**HA-**	SET		37.1	17.9	3.90E-15	9.40E+02	0.996	9.36E+02	0.1
		FHT	O4-H	18.7	8347.5	9.40E+02				
	**A2-**	SET		5.6	15.4	4.30E+08	4.30E+08	0.004	1.72E+06	99.9
	k_{total}								**1.72E+06**	
	$k_{overall}$								**4.30E+03**	
8	**H2A**	SET		43.8	18.1	4.80E-20	1.10E+02	0.001	1.10E-01	0
		FHT	O3-H	19.9	7483	1.10E+02				
	**HA-**	SET		42.7	16.4	3.20E-19	3.10E+02	0.998	3.09E+02	1
		FHT	O3-H	19.4	8490	3.10E+02				
	**A2-**	SET		7.2	14.4	3.20E+07	3.20E+07	0.001	3.20E+04	99
	k_{total}								**3.23E+04**	
	$k_{overall}$								**8.08E+01**	

Table S3: Calculated log(k_{total}) at 298.15 K, in the reactions of the HCA with HOO$^{•}$ in water following pH values

pH	Compounds								
	1	2	3	4	5	6	7	8	Trolox
0	3.204	2.771	3.446	4.415	4.699	3.591	2.196	1.230	4.531
0.1	3.204	2.771	3.446	4.415	4.699	3.591	2.201	1.230	4.531
0.2	3.204	2.771	3.446	4.415	4.699	3.591	2.207	1.230	4.531
0.3	3.204	2.771	3.446	4.415	4.699	3.591	2.215	1.230	4.531
0.4	3.204	2.771	3.446	4.415	4.699	3.591	2.223	1.230	4.531
0.5	3.204	2.771	3.446	4.415	4.699	3.591	2.236	1.230	4.531
0.6	3.204	2.771	3.446	4.415	4.699	3.591	2.250	1.230	4.533
0.7	3.204	2.771	3.446	4.415	4.699	3.591	2.267	1.230	4.533
0.8	3.204	2.771	3.446	4.415	4.699	3.591	2.286	1.230	4.533
0.9	3.204	2.771	3.446	4.415	4.699	3.591	2.310	1.230	4.533
1	3.204	2.771	3.446	4.415	4.699	3.591	2.334	1.230	4.533
1.1	3.204	2.771	3.450	4.415	4.699	3.591	2.364	1.230	4.534
1.2	3.204	2.771	3.450	4.415	4.699	3.616	2.398	1.238	4.534
1.3	3.204	2.771	3.450	4.417	4.699	3.616	2.435	1.238	4.535
1.4	3.204	2.771	3.450	4.417	4.699	3.616	2.473	1.238	4.535
1.5	3.204	2.771	3.450	4.417	4.699	3.616	2.515	1.238	4.536
1.6	3.204	2.771	3.456	4.417	4.703	3.638	2.556	1.238	4.538
1.7	3.204	2.771	3.456	4.417	4.703	3.638	2.600	1.246	4.539
1.8	3.204	2.772	3.461	4.417	4.703	3.638	2.641	1.246	4.541
1.9	3.204	2.772	3.461	4.417	4.703	3.661	2.683	1.253	4.544
2	3.204	2.772	3.465	4.418	4.703	3.681	2.722	1.253	4.547
2.1	3.204	2.772	3.471	4.418	4.708	3.702	2.757	1.260	4.551
2.2	3.204	2.772	3.481	4.418	4.708	3.721	2.790	1.267	4.555

2.3	3.204	2.772	3.486	4.420	4.713	3.757	2.820	1.281	4.561
2.4	3.204	2.772	3.496	4.422	4.713	3.790	2.845	1.286	4.568
2.5	3.204	2.773	3.509	4.425	4.717	3.820	2.867	1.299	4.577
2.6	3.204	2.774	3.522	4.427	4.721	3.863	2.886	1.318	4.588
2.7	3.204	2.775	3.539	4.430	4.725	3.914	2.902	1.336	4.600
2.8	3.204	2.775	3.565	4.433	4.734	3.970	2.915	1.360	4.615
2.9	3.207	2.776	3.588	4.436	4.742	4.029	2.926	1.391	4.633
3	3.207	2.777	3.617	4.442	4.750	4.090	2.936	1.422	4.653
3.1	3.207	2.779	3.651	4.449	4.766	4.161	2.943	1.458	4.676
3.2	3.207	2.781	3.688	4.456	4.781	4.230	2.949	1.501	4.702
3.3	3.207	2.783	3.732	4.465	4.799	4.305	2.954	1.547	4.729
3.4	3.210	2.786	3.777	4.477	4.820	4.380	2.958	1.598	4.759
3.5	3.210	2.790	3.826	4.491	4.844	4.459	2.960	1.651	4.791
3.6	3.210	2.794	3.880	4.507	4.875	4.534	2.963	1.708	4.823
3.7	3.212	2.799	3.936	4.525	4.907	4.611	2.965	1.768	4.855
3.8	3.212	2.804	3.991	4.547	4.947	4.685	2.967	1.831	4.887
3.9	3.215	2.810	4.049	4.571	4.988	4.758	2.968	1.892	4.917
4	3.215	2.817	4.104	4.595	5.033	4.827	2.969	1.954	4.945
4.1	3.217	2.825	4.158	4.622	5.083	4.894	2.970	2.013	4.970
4.2	3.217	2.833	4.210	4.651	5.134	4.954	2.970	2.072	4.993
4.3	3.220	2.841	4.258	4.681	5.190	5.013	2.971	2.127	5.013
4.4	3.223	2.849	4.301	4.711	5.243	5.064	2.972	2.176	5.031
4.5	3.223	2.857	4.342	4.740	5.297	5.111	2.972	2.223	5.046
4.6	3.223	2.865	4.377	4.766	5.350	5.149	2.972	2.265	5.058
4.7	3.225	2.872	4.408	4.792	5.400	5.185	2.972	2.301	5.069

4.8	3.225	2.879	4.435	4.816	5.447	5.217	2.972	2.332	5.077
4.9	3.228	2.884	4.458	4.836	5.491	5.243	2.973	2.360	5.084
5	3.228	2.889	4.477	4.854	5.531	5.265	2.973	2.384	5.090
5.1	3.228	2.893	4.493	4.870	5.567	5.283	2.973	2.403	5.095
5.2	3.228	5.779	4.505	4.884	5.598	5.297	2.973	2.420	5.099
5.3	3.228	5.779	4.517	4.895	5.625	5.310	2.973	2.435	5.102
5.4	3.228	5.779	6.467	4.904	5.648	5.320	2.973	2.446	5.104
5.5	3.230	5.779	6.467	4.912	5.667	5.328	2.973	2.455	5.106
5.6	3.230	6.079	6.467	4.918	5.684	5.334	2.973	2.461	5.108
5.7	3.230	6.079	6.467	4.923	5.698	5.340	2.973	2.467	5.109
5.8	3.230	6.255	6.766	4.927	5.709	5.344	2.973	2.473	5.110
5.9	3.230	6.255	6.766	4.931	5.719	5.348	2.973	2.476	5.111
6	3.230	6.380	6.942	4.933	6.822	5.350	2.973	2.480	5.111
6.1	3.230	6.477	6.942	4.936	6.822	5.352	2.973	2.481	5.112
6.2	5.086	6.623	7.064	6.777	6.822	5.354	2.973	2.483	5.112
6.3	5.086	6.681	7.161	6.777	6.823	5.356	2.973	2.486	5.113
6.4	5.086	6.820	7.307	6.777	7.107	5.358	2.973	2.486	5.113
6.5	5.086	6.892	7.365	6.777	7.107	5.358	2.973	2.487	5.113
6.6	5.086	7.009	7.462	7.076	7.107	5.358	5.634	2.489	5.113
6.7	5.384	7.100	7.576	7.076	7.276	5.360	5.634	2.489	5.113
6.8	5.384	7.193	7.667	7.250	7.398	5.360	5.634	2.490	5.114
6.9	5.559	7.297	7.763	7.250	7.493	5.360	5.634	2.490	5.114
7	5.559	7.391	7.877	7.375	7.571	5.360	5.935	2.490	5.114
7.1	5.683	7.486	7.968	7.471	7.694	5.362	5.935	4.509	5.114
7.2	5.780	7.577	8.064	7.550	7.790	5.362	5.935	4.509	5.114

7.3	5.925	7.670	8.161	7.675	7.868	6.877	6.111	4.509	5.114
7.4	5.983	7.765	8.255	7.813	7.992	6.877	6.236	4.509	5.114
7.5	6.079	7.854	8.348	7.885	8.090	6.877	6.332	4.509	5.114
7.6	6.193	7.940	8.441	7.975	8.167	6.877	6.412	4.808	5.114
7.7	6.283	8.025	8.526	8.072	8.279	7.170	6.537	4.808	5.114
7.8	6.380	8.104	8.615	8.170	8.365	7.170	6.633	4.984	5.114
7.9	6.494	8.182	8.701	8.262	8.467	7.170	6.713	5.107	5.114
8	6.584	8.253	8.780	8.362	8.556	7.344	6.810	5.107	5.114
8.1	6.681	8.320	8.859	8.461	8.655	7.468	6.912	5.283	5.114
8.2	6.778	8.384	8.931	8.556	8.745	7.565	7.013	5.350	5.114
8.3	6.872	8.441	8.999	8.646	8.835	7.643	7.111	5.459	5.114
8.4	6.966	8.491	9.061	8.740	8.922	7.768	7.201	5.547	5.114
8.5	7.057	8.537	9.121	8.828	9.004	7.819	7.305	5.651	5.114
8.6	7.143	8.576	9.173	8.917	9.090	7.943	7.396	5.736	5.114
8.7	7.230	8.612	9.220	9.000	9.164	8.041	7.491	5.848	6.910
8.8	7.318	8.640	9.258	9.079	9.241	8.143	7.583	5.937	6.910
8.9	7.398	8.666	9.294	9.158	9.310	8.225	7.675	6.037	6.910
9	7.476	8.687	9.324	9.233	9.373	8.326	7.760	6.140	6.910
9.1	7.548	8.704	9.352	9.301	9.431	8.431	7.846	6.230	7.208
9.2	7.616	8.719	9.373	9.364	9.484	8.526	7.928	6.324	7.208
9.3	7.679	8.730	9.391	9.422	9.531	8.619	8.004	6.418	7.208
9.4	7.736	8.740	9.408	9.476	9.573	8.715	8.079	6.509	7.383
9.5	7.788	8.747	9.422	9.521	9.609	8.803	8.149	6.599	7.507
9.6	7.834	8.753	9.431	9.562	9.639	8.893	8.215	6.684	7.603
9.7	7.874	8.758	9.442	9.598	9.667	8.980	8.274	6.768	7.682

9.8	7.909	8.762	9.452	9.628	9.688	9.068	8.326	6.848	7.807
9.9	7.940	8.766	9.459	9.654	9.707	9.149	8.375	6.923	7.858
10	7.965	8.768	9.470	9.676	9.721	9.228	8.417	6.995	7.983
10.1	7.986	8.770	9.477	9.694	9.734	9.303	8.453	7.061	8.080
10.2	8.004	8.772	9.487	9.708	9.744	9.375	8.486	7.124	8.182
10.3	8.017	8.773	9.497	9.720	9.752	9.438	8.512	7.179	8.265
10.4	8.029	8.775	9.509	9.730	9.759	9.498	8.534	7.228	8.366
10.5	8.041	8.775	9.521	9.739	9.764	9.551	8.553	7.272	8.471
10.6	8.045	8.775	9.535	9.745	9.769	9.601	8.568	7.312	8.566
10.7	8.053	8.776	9.551	9.750	9.772	9.642	8.581	7.344	8.659
10.8	8.057	8.777	9.571	9.754	9.775	9.680	8.591	7.373	8.754
10.9	8.064	8.777	9.590	9.757	9.777	9.712	8.600	7.398	8.843
11	8.068	8.777	9.612	9.760	9.778	9.739	8.606	7.417	8.933
11.1	8.068	8.777	9.634	9.763	9.780	9.762	8.612	7.435	9.020
11.2	8.072	8.777	9.659	9.764	9.781	9.781	8.616	7.447	9.107
11.3	8.072	8.777	9.683	9.766	9.782	9.797	8.620	7.459	9.189
11.4	8.076	8.777	9.708	9.766	9.782	9.810	8.622	7.468	9.269
11.5	8.076	8.777	9.732	9.767	9.783	9.820	8.624	7.476	9.342
11.6	8.076	8.777	9.755	9.768	9.784	9.829	8.626	7.481	9.414
11.7	8.076	8.778	9.776	9.769	9.784	9.836	8.627	7.486	9.478
11.8	8.076	8.778	9.794	9.769	9.784	9.841	8.629	7.490	9.538
11.9	8.079	8.778	9.812	9.769	9.785	9.846	8.630	7.493	9.592
12	8.079	8.778	9.826	9.770	9.785	9.849	8.630	7.496	9.640
12.1	8.079	8.778	9.839	9.770	9.785	9.852	8.631	7.498	9.683
12.2	8.079	8.778	9.849	9.770	9.785	9.854	8.631	7.500	9.720

12.3	8.079	8.778	9.859	9.770	9.785	9.856	8.632	7.500	9.752
12.4	8.079	8.778	9.866	9.770	9.785	9.858	8.632	7.501	9.779
12.5	8.079	8.778	9.872	9.770	9.785	9.859	8.632	7.502	9.802
12.6	8.079	8.778	9.877	9.770	9.785	9.860	8.632	7.502	9.821
12.7	8.079	8.778	9.881	9.771	9.785	9.860	8.633	7.504	9.837
12.8	8.079	8.778	9.884	9.771	9.785	9.861	8.633	7.504	9.850
12.9	8.079	8.778	9.887	9.771	9.785	9.862	8.633	7.504	9.860
13	8.079	8.778	9.889	9.771	9.785	9.862	8.633	7.504	9.868
13.1	8.079	8.778	9.891	9.771	9.785	9.862	8.633	7.504	9.875
13.2	8.079	8.778	9.892	9.771	9.785	9.863	8.633	7.505	9.881
13.3	8.079	8.778	9.893	9.771	9.785	9.863	8.633	7.505	9.885
13.4	8.079	8.778	9.894	9.771	9.785	9.863	8.633	7.505	9.889
13.5	8.079	8.778	9.895	9.771	9.785	9.863	8.633	7.505	9.892
13.6	8.079	8.778	9.895	9.771	9.785	9.863	8.633	7.505	9.894
13.7	8.079	8.778	9.896	9.771	9.785	9.863	8.633	7.505	9.896
13.8	8.079	8.778	9.897	9.771	9.785	9.863	8.633	7.505	9.897
13.9	8.079	8.778	9.897	9.771	9.785	9.863	8.633	7.505	9.899
14	8.079	8.778	9.897	9.771	9.785	9.863	8.633	7.505	9.900

Table S4: Calculated log($k_{overall}$) at 298.15 K, in the reactions of the HCA with HOO• in water following pH values

pH	Compounds								
	**1**	**2**	**3**	**4**	**5**	**6**	**7**	**8**	Trolox
0	3.204	2.771	3.446	4.415	4.699	3.591	2.196	1.230	4.531
0.1	3.204	2.771	3.446	4.415	4.699	3.591	2.201	1.230	4.531
0.2	3.204	2.771	3.446	4.415	4.699	3.591	2.207	1.230	4.531
0.3	3.204	2.771	3.446	4.415	4.699	3.591	2.215	1.230	4.531
0.4	3.204	2.771	3.446	4.415	4.699	3.591	2.223	1.230	4.531
0.5	3.204	2.771	3.446	4.415	4.699	3.591	2.235	1.230	4.531
0.6	3.204	2.771	3.446	4.415	4.699	3.591	2.250	1.230	4.533
0.7	3.204	2.771	3.446	4.415	4.699	3.591	2.267	1.230	4.533
0.8	3.204	2.771	3.446	4.415	4.699	3.591	2.286	1.230	4.533
0.9	3.204	2.771	3.446	4.415	4.699	3.591	2.310	1.230	4.533
1	3.204	2.771	3.446	4.415	4.699	3.591	2.334	1.230	4.533
1.1	3.204	2.771	3.450	4.415	4.699	3.591	2.364	1.230	4.534
1.2	3.204	2.771	3.450	4.415	4.699	3.616	2.398	1.238	4.534
1.3	3.204	2.771	3.450	4.417	4.699	3.616	2.434	1.238	4.535
1.4	3.204	2.771	3.450	4.416	4.699	3.616	2.473	1.238	4.535
1.5	3.204	2.771	3.450	4.416	4.699	3.616	2.514	1.238	4.536
1.6	3.204	2.771	3.456	4.416	4.703	3.638	2.556	1.238	4.537
1.7	3.204	2.771	3.456	4.416	4.703	3.638	2.600	1.245	4.538
1.8	3.204	2.771	3.460	4.416	4.703	3.638	2.641	1.245	4.541
1.9	3.204	2.771	3.460	4.416	4.703	3.660	2.682	1.252	4.543
2	3.203	2.771	3.465	4.418	4.703	3.681	2.721	1.252	4.546
2.1	3.203	2.771	3.470	4.417	4.707	3.701	2.757	1.259	4.550

2.2	3.203	2.771	3.480	4.417	4.706	3.720	2.789	1.266	4.554
2.3	3.203	2.771	3.484	4.419	4.711	3.755	2.818	1.280	4.560
2.4	3.202	2.771	3.494	4.420	4.711	3.788	2.843	1.284	4.566
2.5	3.202	2.771	3.507	4.423	4.715	3.818	2.865	1.297	4.575
2.6	3.201	2.771	3.520	4.424	4.718	3.860	2.883	1.315	4.585
2.7	3.201	2.771	3.536	4.426	4.722	3.910	2.899	1.333	4.597
2.8	3.200	2.771	3.560	4.429	4.730	3.966	2.911	1.356	4.611
2.9	3.201	2.771	3.582	4.431	4.737	4.024	2.921	1.386	4.627
3	3.200	2.771	3.610	4.436	4.743	4.083	2.929	1.415	4.646
3.1	3.198	2.770	3.643	4.440	4.757	4.153	2.934	1.449	4.668
3.2	3.196	2.770	3.678	4.446	4.770	4.220	2.938	1.490	4.691
3.3	3.193	2.770	3.718	4.452	4.786	4.292	2.940	1.533	4.715
3.4	3.193	2.769	3.760	4.460	4.803	4.363	2.941	1.581	4.742
3.5	3.188	2.768	3.805	4.470	4.823	4.438	2.939	1.630	4.769
3.6	3.183	2.767	3.853	4.480	4.848	4.507	2.937	1.681	4.797
3.7	3.179	2.765	3.902	4.492	4.874	4.577	2.932	1.735	4.822
3.8	3.171	2.763	3.950	4.505	4.906	4.643	2.926	1.789	4.845
3.9	3.163	2.759	3.998	4.519	4.937	4.707	2.917	1.840	4.865
4	3.151	2.753	4.040	4.532	4.970	4.763	2.905	1.890	4.881
4.1	3.138	2.746	4.079	4.543	5.004	4.815	2.891	1.934	4.891
4.2	3.120	2.735	4.112	4.554	5.036	4.857	2.873	1.975	4.896
4.3	3.101	2.721	4.138	4.562	5.071	4.893	2.852	2.008	4.894
4.4	3.077	2.703	4.156	4.565	5.098	4.919	2.826	2.031	4.885
4.5	3.046	2.680	4.166	4.563	5.120	4.934	2.796	2.046	4.869
4.6	3.010	2.652	4.164	4.554	5.138	4.937	2.760	2.052	4.846

4.7	2.971	2.618	4.154	4.538	5.146	4.931	2.718	2.047	4.815
4.8	2.924	2.577	4.134	4.515	5.146	4.916	2.671	2.031	4.776
4.9	2.874	2.530	4.104	4.482	5.137	4.889	2.619	2.006	4.731
5	2.815	2.477	4.065	4.442	5.119	4.852	2.560	1.971	4.678
5.1	2.752	2.417	4.016	4.393	5.091	4.807	2.496	1.927	4.619
5.2	2.682	5.233	3.960	4.338	5.052	4.751	2.427	1.874	4.553
5.3	2.609	5.160	3.898	4.276	5.006	4.690	2.353	1.815	4.483
5.4	2.531	5.082	5.770	4.207	4.951	4.623	2.276	1.748	4.407
5.5	2.451	5.000	5.688	4.133	4.888	4.549	2.194	1.676	4.327
5.6	2.367	5.215	5.603	4.054	4.820	4.471	2.109	1.597	4.244
5.7	2.279	5.128	5.515	3.972	4.747	4.389	2.022	1.515	4.158
5.8	2.189	5.214	5.725	3.886	4.668	4.303	1.932	1.431	4.069
5.9	2.097	5.122	5.633	3.798	4.585	4.215	1.840	1.343	3.978
6	2.004	5.154	5.715	3.707	5.595	4.124	1.747	1.254	3.885
6.1	1.909	5.156	5.620	3.614	5.501	4.031	1.652	1.160	3.791
6.2	3.670	5.206	5.648	5.361	5.405	3.937	1.556	1.066	3.696
6.3	3.573	5.168	5.649	5.265	5.310	3.843	1.460	0.973	3.600
6.4	3.476	5.209	5.697	5.167	5.496	3.747	1.362	0.875	3.502
6.5	3.379	5.184	5.658	5.070	5.399	3.650	1.265	0.779	3.406
6.6	3.279	5.202	5.656	5.269	5.300	3.551	3.828	0.682	3.306
6.7	3.477	5.194	5.670	5.169	5.370	3.453	3.728	0.582	3.207
6.8	3.379	5.189	5.662	5.246	5.394	3.355	3.630	0.486	3.109
6.9	3.456	5.194	5.661	5.148	5.390	3.257	3.532	0.388	3.011
7	3.358	5.190	5.677	5.174	5.370	3.159	3.734	0.289	2.913
7.1	3.382	5.185	5.667	5.170	5.393	3.061	3.634	2.208	2.813

7.2	3.382	5.180	5.667	5.152	5.392	2.964	3.537	2.111	2.716
7.3	3.430	5.175	5.667	5.180	5.373	4.382	3.616	2.014	2.619
7.4	3.381	5.163	5.653	5.211	5.390	4.275	3.633	1.907	2.512
7.5	3.380	5.155	5.649	5.186	5.391	4.178	3.633	1.810	2.415
7.6	3.397	5.144	5.645	5.180	5.371	4.081	3.616	2.012	2.318
7.7	3.397	5.139	5.640	5.186	5.393	4.284	3.651	1.922	2.228
7.8	3.380	5.104	5.615	5.170	5.365	4.170	3.633	1.984	2.114
7.9	3.397	5.085	5.604	5.166	5.370	4.073	3.616	2.010	2.017
8	3.362	5.031	5.558	5.140	5.334	4.123	3.588	1.885	1.892
8.1	3.380	5.019	5.558	5.160	5.354	4.167	3.611	1.982	1.813
8.2	3.380	4.986	5.533	5.158	5.347	4.167	3.615	1.952	1.716
8.3	3.349	4.918	5.476	5.124	5.312	4.121	3.588	1.937	1.591
8.4	3.443	4.968	5.538	5.217	5.399	4.245	3.679	2.024	1.591
8.5	3.358	4.838	5.422	5.129	5.305	4.120	3.606	1.952	1.415
8.6	3.444	4.877	5.474	5.218	5.391	4.245	3.697	2.037	1.415
8.7	3.230	4.612	5.220	5.000	5.164	4.041	3.491	1.848	2.910
8.8	3.318	4.640	5.258	5.079	5.241	4.143	3.583	1.937	2.910
8.9	3.398	4.666	5.294	5.158	5.310	4.225	3.675	2.037	2.910
9	3.476	4.687	5.324	5.233	5.373	4.326	3.760	2.140	2.910
9.1	3.548	4.704	5.352	5.301	5.431	4.431	3.846	2.230	3.208

Table S5: The Cartesian coordinates and energies of TS of the reaction between HCA with HOO• following the FHT mechanism in studied solvents

Name				TS-1-O2-H-OOH (pentyl ethanoate)
Cartesian Coordinates				Frequency and Energy
O	-0.83281400	2.03341600	-0.04963000	Zero-point correction= 0.165319 (Hartree/Particle)
O	3.79030700	0.91809800	0.25260400	Thermal correction to Energy= 0.178912
O	3.31051600	-1.23981600	-0.07946800	Thermal correction to Enthalpy= 0.179857
C	-0.89146200	-0.35997800	0.06367100	Thermal correction to Gibbs Free Energy= 0.122457
C	-1.52735500	0.92108500	0.01644100	Sum of electronic and zero-point Energies= -724.117669
C	-1.70522500	-1.49601500	0.13501200	Sum of electronic and thermal Energies= -724.104075
C	-2.93941300	1.00058100	0.05085200	Sum of electronic and thermal Enthalpies= -724.103131
C	0.55232100	-0.55775800	0.02921900	Sum of electronic and thermal Free Energies= -724.160531
C	-3.08790200	-1.39716600	0.18416800	
C	-3.70718700	-0.13977300	0.14583000	
C	1.53019100	0.35068300	0.15183700	
C	2.93592600	-0.10592400	0.09075800	
H	-1.23333400	-2.47224300	0.15731500	
H	-3.38621400	1.98736900	0.01419600	
H	0.86251600	-1.59296100	-0.09534700	
H	-3.69011200	-2.29542100	0.24901000	
H	-4.78751000	-0.06596500	0.18442800	
H	1.36170100	1.40520000	0.30920800	
H	-0.37665400	2.12882200	-1.07925600	
H	4.69174800	0.56555900	0.20287400	
H	-1.57515700	1.07406900	-2.99480300	
O	-0.14210700	2.04351900	-2.30101400	
O	-0.70150900	0.84916900	-2.63061300	
**Name**				**TS-1-O2-H-OOH (water)**
Cartesian Coordinates				Frequency and Energy
O	-0.83031400	2.03393000	0.00852300	Zero-point correction= 0.165797 (Hartree/Particle)
O	3.80451100	0.90903800	0.10777300	Thermal correction to Energy= 0.179012
O	3.31061200	-1.27404100	0.06295800	Thermal correction to Enthalpy= 0.179957
C	-0.88709800	-0.36419600	0.09608100	Thermal correction to Gibbs Free Energy= 0.124278
C	-1.52789100	0.91385900	0.03213100	Sum of electronic and zero-point Energies= -724.121250
C	-1.69867900	-1.50268800	0.14869500	Sum of electronic and thermal Energies= -724.108035
C	-2.93907400	0.99052600	0.00864500	Sum of electronic and thermal Enthalpies= -724.107090
C	0.55590300	-0.56442300	0.09285600	Sum of electronic and thermal Free Energies= -724.162769
C	-3.08305900	-1.40753200	0.14568800	
C	-3.70525100	-0.15268000	0.07555900	
C	1.54113700	0.34662600	0.10736900	
C	2.94172300	-0.11804800	0.09052900	
H	-1.22441300	-2.47669500	0.19165200	
H	-3.38820100	1.97498000	-0.04942000	
H	0.85557400	-1.60984500	0.07879200	
H	-3.68436900	-2.30716000	0.19220900	
H	-4.78619800	-0.08327700	0.06860800	
H	1.38656900	1.41405400	0.13962400	
H	-0.38868200	2.16456600	-1.01799500	
H	4.71024500	0.56051600	0.09508400	
H	-1.64655700	1.14886600	-2.84311000	
O	-0.12269100	2.05941400	-2.24448100	
O	-0.74252500	0.89876100	-2.58047600	
**Name**				**TS-1-ANION-O2-H-OOH (water)**
Cartesian Coordinates				Frequency and Energy

O -0.85502400 2.05018300 -0.03189600	Zero-point correction= 0.152393 (Hartree/Particle)
O 3.83237500 0.87919600 0.15948000	Thermal correction to Energy= 0.165572
O 3.27057500 -1.28278100 0.05840600	Thermal correction to Enthalpy= 0.166516
C -0.89267600 -0.34964800 0.08303700	Thermal correction to Gibbs Free Energy= 0.110446
C -1.54413400 0.92205400 0.01313100	Sum of electronic and zero-point Energies= -723.678788
C -1.70430700 -1.49171800 0.14423800	Sum of electronic and thermal Energies= -723.665609
C -2.95604800 0.99330700 0.00304500	Sum of electronic and thermal Enthalpies= -723.664665
C 0.55266700 -0.54527400 0.07804000	Sum of electronic and thermal Free Energies= -723.720734
C -3.08646400 -1.40369300 0.15316500	
C -3.71698800 -0.15060000 0.08432600	
C 1.54050200 0.36014500 0.14373000	
C 2.99094500 -0.05882300 0.11939200	
H -1.22517300 -2.46339500 0.18848400	
H -3.40904400 1.97582500 -0.05889500	
H 0.85280000 -1.58910900 0.02394500	
H -3.68339700 -2.30591100 0.20896700	
H -4.79831200 -0.08629000 0.08774900	
H 1.36126500 1.42313400 0.22457500	
H -0.36733100 2.13149500 -1.02526000	
H -1.61971600 1.13081100 -2.87568400	
O -0.07394500 2.00030000 -2.26938000	
O -0.72754100 0.85482800 -2.60038400	
**Name**	**TS-2-O4-H-OOH (pentyl ethanoate)**
Cartesian Coordinates	Frequency and Energy
O -4.37734400 -0.66704900 0.00056500	Zero-point correction= 0.165623 (Hartree/Particle)
O 4.37474400 -0.91841100 -0.21150800	Thermal correction to Energy= 0.179266
O 3.83254800 1.23706500 0.03887100	Thermal correction to Enthalpy= 0.180211
C -0.33423400 0.12824700 0.02993800	Thermal correction to Gibbs Free Energy= 0.122045
C -1.26266600 1.17446500 0.15938800	Sum of electronic and zero-point Energies= -724.118771
C -0.80984400 -1.19642000 -0.08963500	Sum of electronic and thermal Energies= -724.105127
C -2.61951900 0.92443400 0.16550900	Sum of electronic and thermal Enthalpies= -724.104183
C -2.15700300 -1.46249100 -0.07573600	Sum of electronic and thermal Free Energies= -724.162349
C -3.09322000 -0.40366800 0.03906800	
C 1.08673600 0.45540900 0.02602700	
C 2.10164300 -0.41108500 -0.09469500	
C 3.49191300 0.08608500 -0.07784100	
H -0.90068300 2.19227900 0.25471000	
H -0.10756900 -2.01523900 -0.18964600	
H -3.34018200 1.72813600 0.26116300	
H -2.53561200 -2.47372600 -0.16683700	
H 1.33446800 1.50910900 0.13127000	
H 1.96434800 -1.47950900 -0.20637900	
H -4.87464200 -0.32477900 0.94745700	
H 5.26600200 -0.53771200 -0.19524900	
H -3.64417300 -0.46780400 3.18364500	
O -5.14864000 -0.10366500 2.14712700	
O -3.95509400 0.29552700 2.66819300	
**Name**	**TS-2-O4-H-OOH (water)**
Cartesian Coordinates	Frequency and Energy
O -4.46174400 -0.31988200 -0.00431800	Zero-point correction= 0.165056 (Hartree/Particle)
O 4.28733700 -0.96764300 0.03749100	Thermal correction to Energy= 0.178569
O 3.82478100 1.21832000 -0.10865600	Thermal correction to Enthalpy= 0.179513
C -0.37905000 0.27435300 -0.01038800	Thermal correction to Gibbs Free Energy= 0.122034
C -1.26352600 1.36782300 -0.09595900	Sum of electronic and zero-point Energies= -724.123065

C -0.90579900 -1.02769800 0.10153900	Sum of electronic and thermal Energies= -724.109552
C -2.62801600 1.18082300 -0.06950900	Sum of electronic and thermal Enthalpies= -724.108608
C -2.26668000 -1.22969000 0.11929000	Sum of electronic and thermal Free Energies= -724.166087
C -3.15227800 -0.12675300 0.02517700	
C 1.05321600 0.54637300 -0.04158500	
C 2.03341600 -0.36500900 0.02774800	
C 3.44023500 0.07035900 -0.02237200	
H -0.85463200 2.36848100 -0.18045900	
H -0.24227300 -1.88091300 0.17117900	
H -3.31696600 2.01456500 -0.13305600	
H -2.68797700 -2.22550100 0.20040200	
H 1.33277400 1.59304300 -0.13154900	
H 1.85669000 -1.42950200 0.11675100	
H -4.81233000 -0.58885700 1.02130000	
H 5.19780500 -0.63334500 -0.00025000	
H -3.19227600 -1.33296500 2.73776100	
O -4.91181200 -0.75836100 2.27161600	
O -3.65891900 -0.47882500 2.71325100	
**Name**	**TS-2-ANION-O4-H-OOH (water)**
Cartesian Coordinates	Frequency and Energy
O -3.61605600 0.40712600 -0.97728900	Zero-point correction= 0.151482 (Hartree/Particle)
O 5.01378600 -1.19778100 -0.17392200	Thermal correction to Energy= 0.164226
O 4.61289800 0.79747400 0.75349800	Thermal correction to Enthalpy= 0.165171
C 0.44408400 0.43000800 -0.18210200	Thermal correction to Gibbs Free Energy= 0.108740
C -0.36834000 1.51946700 0.18810300	Sum of electronic and zero-point Energies= -723.681327
C -0.15489300 -0.67951600 -0.81410600	Sum of electronic and thermal Energies= -723.668583
C -1.72525900 1.51318200 -0.05515900	Sum of electronic and thermal Enthalpies= -723.667638
C -1.50750100 -0.69669300 -1.06758300	Sum of electronic and thermal Free Energies= -723.724069
C -2.31557100 0.40446900 -0.69666500	
C 1.87417200 0.50353300 0.10903200	
C 2.79202700 -0.43072400 -0.16973600	
C 4.25083900 -0.25430400 0.16790300	
H 0.08969600 2.37281200 0.67585400	
H 0.44954200 -1.52910700 -1.10757300	
H -2.35609500 2.34689100 0.23014100	
H -1.97825800 -1.54276700 -1.55545300	
H 2.20441100 1.41527000 0.59992200	
H 2.53262000 -1.36332000 -0.65975500	
H -4.14501500 -0.16744500 -0.20508900	
H -2.85104500 -1.72089200 1.23029400	
O -4.45443500 -0.82628900 0.86755700	
O -3.26659200 -0.88902700 1.51934600	
**Name**	**TS-3-O3-H-OOH (pentyl ethanoate)**
Cartesian Coordinates	Frequency and Energy
O -3.37014100 1.68826000 -0.01861600	Zero-point correction= 0.170550 (Hartree/Particle)
O -4.12186500 -0.89546500 0.07535100	Thermal correction to Energy= 0.185102
O 4.65502500 -0.81786100 -0.08919100	Thermal correction to Enthalpy= 0.186046
O 4.03133400 1.32727100 0.00664000	Thermal correction to Gibbs Free Energy= 0.126329
C -0.09586300 0.07113500 0.05645500	Sum of electronic and zero-point Energies= -799.347649
C -1.05064000 1.07717200 0.02100400	Sum of electronic and thermal Energies= -799.333097
C -0.52794000 -1.27734200 0.10621100	Sum of electronic and thermal Enthalpies= -799.332153
C -2.42040800 0.76763900 0.02820000	Sum of electronic and thermal Free Energies= -799.391869
C -2.82495400 -0.59382600 0.07331900	
C -1.86593600 -1.60856300 0.11237400	

<table>
<tr><td>C 1.31827200 0.44303800 0.03452600
C 2.36358300 -0.39072200 -0.01017600
C 3.73279400 0.15886500 -0.02795200
H -0.75994400 2.12199300 -0.02263400
H 0.20540300 -2.07416200 0.14331900
H -2.19215600 -2.64050600 0.15187100
H 1.52680700 1.51043200 0.04942400
H 2.26738100 -1.46888000 -0.04082000
H -3.52430000 2.08048500 1.00139300
H -4.62561000 -0.06756500 0.01722200
H 5.53013600 -0.40144800 -0.09930700
H -1.98314700 1.25980400 2.78341600
O -3.51810300 2.19878600 2.28583800
O -2.92659200 1.04351100 2.67844400</td><td></td></tr>
<tr><td>Name</td><td>TS-3-O4-H-OOH (pentyl ethanoate)</td></tr>
<tr><td>Cartesian Coordinates</td><td>Frequency and Energy</td></tr>
<tr><td>O -3.33068500 1.68609400 0.03450400
O -4.12416400 -0.87650600 -0.00211900
O 4.65053800 -0.85146300 -0.13026600
O 4.02329400 1.28317900 0.09612000
C -0.09979400 0.01279200 0.05442300
C -1.05368900 1.03162600 0.05197200
C -0.51466400 -1.34014100 0.04750300
C -2.40499100 0.71554200 0.03609700
C -2.82236500 -0.64068800 0.03193800
C -1.85024300 -1.65998500 0.03730200
C 1.31141600 0.39631100 0.06206800
C 2.35785100 -0.43167200 -0.03825300
C 3.72854600 0.11907600 -0.01333000
H -0.75263000 2.07312400 0.05653400
H 0.22248100 -2.13300300 0.05822600
H -2.18732900 -2.68997700 0.03857800
H 1.51645800 1.46063900 0.14974800
H 2.26250500 -1.50520700 -0.14284100
H -4.20028200 1.26791300 -0.06609700
H -4.47447900 -1.08960700 1.01326500
H 5.52629800 -0.43656100 -0.10886100
H -3.37919800 0.18481500 2.97371200
O -4.59629800 -1.06116200 2.31144300
O -3.33744700 -0.74938800 2.70293900</td><td>Zero-point correction= 0.170792 (Hartree/Particle)
Thermal correction to Energy= 0.185369
Thermal correction to Enthalpy= 0.186313
Thermal correction to Gibbs Free Energy= 0.126351
Sum of electronic and zero-point Energies= -799.347096
Sum of electronic and thermal Energies= -799.332519
Sum of electronic and thermal Enthalpies= -799.331575
Sum of electronic and thermal Free Energies= -799.391537</td></tr>
<tr><td>Name</td><td>TS-3-O3-H-OOH (water)</td></tr>
<tr><td>Cartesian Coordinates</td><td>Frequency and Energy</td></tr>
<tr><td>O -3.28707000 1.79257700 -0.03365100
O -4.08724000 -0.80324600 0.17489900
O 4.67779200 -0.88118300 -0.08226300
O 4.10906900 1.28449000 -0.12646700
C -0.04745600 0.11039700 0.03665900
C -0.98604100 1.12993900 -0.00170000
C -0.49655100 -1.23143300 0.12867100
C -2.35832000 0.84176100 0.03165200
C -2.78262200 -0.51101900 0.12663900
C -1.83811900 -1.53970100 0.17523300
C 1.36996000 0.46246400 -0.01889700
C 2.40014500 -0.39277000 -0.02411300</td><td>Zero-point correction= 0.169697 (Hartree/Particle)
Thermal correction to Energy= 0.184301
Thermal correction to Enthalpy= 0.185245
Thermal correction to Gibbs Free Energy= 0.125585
Sum of electronic and zero-point Energies= -799.355125
Sum of electronic and thermal Energies= -799.340521
Sum of electronic and thermal Enthalpies= -799.339576
Sum of electronic and thermal Free Energies= -799.399236</td></tr>
</table>

C 3.77840600 0.11649300 -0.08261200<br>H -0.68051200 2.16846800 -0.06824400<br>H 0.22412700 -2.03937400 0.16650700<br>H -2.17991600 -2.56460600 0.24937500<br>H 1.58672100 1.52716500 -0.06257200<br>H 2.28272000 -1.46840100 0.01193100<br>H -3.67663800 1.95940900 0.97445100<br>H -4.60361500 0.01646400 0.10809100<br>H 5.57050900 -0.50265300 -0.11830400<br>H -2.21143600 1.29204100 2.86357400<br>O -3.88727000 1.87741400 2.26808200<br>O -3.05350900 0.86735300 2.61937000	
**Name**	**TS-3-O4-H-OOH (water)**
Cartesian Coordinates	Frequency and Energy
O -3.33669800 1.58620300 0.18452700<br>O -4.09445400 -1.02655100 -0.03973900<br>O 4.67213100 -0.79797200 -0.23833200<br>O 4.01250000 1.30239600 0.16985500<br>C -0.08609200 -0.04605000 0.10518700<br>C -1.05711200 0.95199500 0.16951900<br>C -0.47652800 -1.40445100 0.01786800<br>C -2.40392300 0.61591900 0.12655500<br>C -2.79753800 -0.74456300 0.03439900<br>C -1.80674900 -1.74489600 -0.00763400<br>C 1.31662700 0.36604900 0.12298000<br>C 2.37637500 -0.43451300 -0.04470300<br>C 3.73478000 0.13591100 -0.01907100<br>H -0.77449800 1.99618600 0.24024100<br>H 0.27375900 -2.18385800 -0.02272600<br>H -2.12664900 -2.77816500 -0.07024700<br>H 1.49435700 1.42814700 0.27269700<br>H 2.30054900 -1.50097600 -0.21520100<br>H -4.21611600 1.19455400 0.06428200<br>H -4.53564000 -0.93799500 0.95369600<br>H 5.54685500 -0.37778100 -0.22089000<br>H -3.35915500 0.43570600 2.74661700<br>O -4.72346800 -0.73819200 2.24013000<br>O -3.46218600 -0.53135300 2.68459300	Zero-point correction= 0.169952 (Hartree/Particle)<br>Thermal correction to Energy= 0.184395<br>Thermal correction to Enthalpy= 0.185339<br>Thermal correction to Gibbs Free Energy= 0.126490<br>Sum of electronic and zero-point Energies= -799.354450<br>Sum of electronic and thermal Energies= -799.340006<br>Sum of electronic and thermal Enthalpies= -799.339062<br>Sum of electronic and thermal Free Energies= -799.397912
**Name**	**TS-3-ANION-O3-H-OOH (water)**
Cartesian Coordinates	Frequency and Energy
O -3.28827700 1.80184800 -0.02468200<br>O -4.10018500 -0.79355100 0.17726100<br>O 4.71376100 -0.83551300 -0.03678500<br>O 4.07636800 1.30305600 -0.17957500<br>C -0.04968500 0.10783600 0.02840700<br>C -0.98821800 1.12734500 -0.00670900<br>C -0.50646700 -1.23156900 0.11883800<br>C -2.36078600 0.84404000 0.03395500<br>C -2.79155800 -0.50493200 0.12714200<br>C -1.84986100 -1.53577300 0.16912300<br>C 1.37419700 0.45666400 -0.03208400<br>C 2.40697900 -0.39117400 -0.00241000<br>C 3.83840100 0.07173300 -0.07809500<br>H -0.68115600 2.16547500 -0.07407300	Zero-point correction= 0.156846 (Hartree/Particle)<br>Thermal correction to Energy= 0.171234<br>Thermal correction to Enthalpy= 0.172178<br>Thermal correction to Gibbs Free Energy= 0.113018<br>Sum of electronic and zero-point Energies= -798.911588<br>Sum of electronic and thermal Energies= -798.897201<br>Sum of electronic and thermal Enthalpies= -798.896257<br>Sum of electronic and thermal Free Energies= -798.955416

H 0.21117000 -2.04251400 0.14896700<br>H -2.19430100 -2.56022000 0.23939800<br>H 1.58515700 1.52033600 -0.11015400<br>H 2.26639200 -1.46428900 0.07544100<br>H -3.66613800 1.96811600 0.97476900<br>H -4.61198200 0.02875200 0.11245600<br>H -2.20071700 1.25149100 2.85542200<br>O -3.87316600 1.87890500 2.29298900<br>O -3.05730400 0.84864000 2.62501400	
**Name**	**TS-3- ANION-O4-H-OOH (water)**
Cartesian Coordinates	Frequency and Energy
O -3.36120600 1.61348000 0.18018800<br>O -4.13298600 -0.99316600 -0.00169700<br>O 4.67608200 -0.80873900 0.03181400<br>O 3.97868500 1.31505400 0.12420800<br>C -0.10898400 -0.02239000 0.02232500<br>C -1.08055200 0.97644500 0.09441900<br>C -0.50927700 -1.37836200 -0.06025000<br>C -2.42788300 0.64272800 0.09193500<br>C -2.82778400 -0.71489700 0.01889200<br>C -1.84150600 -1.71556100 -0.05704100<br>C 1.29933700 0.38375700 0.04368300<br>C 2.35862800 -0.43218100 0.02373400<br>C 3.77819600 0.07543100 0.06208300<br>H -0.79540600 2.02057900 0.15735300<br>H 0.23592600 -2.16095300 -0.12393400<br>H -2.16413000 -2.74849100 -0.11472700<br>H 1.47590600 1.45539600 0.08830800<br>H 2.25085600 -1.51097400 -0.01662500<br>H -4.24175100 1.21059000 0.11915400<br>H -4.50135600 -0.99452400 1.00928000<br>H -3.24796500 0.37959400 2.76131200<br>O -4.58612300 -0.85978900 2.34699000<br>O -3.30243600 -0.58949700 2.67408900	Zero-point correction= 0.156514 (Hartree/Particle)<br>Thermal correction to Energy= 0.171096<br>Thermal correction to Enthalpy= 0.172040<br>Thermal correction to Gibbs Free Energy= 0.111608<br>Sum of electronic and zero-point Energies= -798.912435<br>Sum of electronic and thermal Energies= -798.897853<br>Sum of electronic and thermal Enthalpies= -798.896909<br>Sum of electronic and thermal Free Energies= -798.957341
**Name**	**TS-3-DIANION-O3-H-OOH (water)**
Cartesian Coordinates	Frequency and Energy
C 0.15679600 1.87834800 0.04422700<br>C 0.77339000 0.60471900 -0.02857700<br>C -0.05550900 -0.49771600 -0.22463800<br>C -1.44673800 -0.38688300 -0.37395700<br>C -2.07588700 0.92418200 -0.30448400<br>C -1.20513200 2.02957500 -0.08258900<br>O -2.15422000 -1.46901800 -0.63696000<br>O -3.34217700 1.07552700 -0.45182900<br>C 2.23599200 0.52889800 0.09796500<br>C 3.03245000 -0.53861900 -0.03211300<br>H 0.78819900 2.74808800 0.20271400<br>H 0.36248800 -1.49663300 -0.28510900<br>H -1.67149000 3.00837300 -0.03023500<br>H -3.19296600 -1.47057700 -0.30171100<br>H 2.74614300 1.46687200 0.31423200<br>H 2.63220800 -1.52427500 -0.25815600<br>H -4.05992000 0.22515500 0.51671400<br>O -4.39909200 -1.59629300 0.23995200	Zero-point correction= 0.151794 (Hartree/Particle)<br>Thermal correction to Energy= 0.164703<br>Thermal correction to Enthalpy= 0.165647<br>Thermal correction to Gibbs Free Energy= 0.109315<br>Sum of electronic and zero-point Energies= -798.459867<br>Sum of electronic and thermal Energies= -798.446958<br>Sum of electronic and thermal Enthalpies= -798.446014<br>Sum of electronic and thermal Free Energies= -798.502346

O -4.48709400 -0.51929600 1.08332200<br>C 4.55915900 -0.46869200 0.11798500<br>O 5.14474200 -1.56669600 -0.03332400<br>O 5.05636700 0.65079200 0.36866700	
**Name**	**TS-4-O4-H-OOH (pentyl ethanoate)**
Cartesian Coordinates	Frequency and Energy
O 3.23028500 0.85872800 -0.03166100<br>O 3.61729000 -1.76278400 -0.09676500<br>O -5.07811900 -0.54628100 -0.14156600<br>O -4.15532700 1.48356700 0.02773900<br>C -0.24655300 -0.33268800 -0.03453700<br>C 2.13860400 0.09445900 -0.03197900<br>C 0.82808900 0.56164500 -0.00200500<br>C 0.00034500 -1.72230200 -0.08285700<br>C 2.38743800 -1.31288100 -0.06739400<br>C 1.28954400 -2.19794900 -0.08436100<br>C -1.59560600 0.22759300 -0.01757800<br>C -2.74821200 -0.45042800 -0.08716900<br>C 3.07354000 2.25086600 0.22068300<br>C -4.02675400 0.28721500 -0.05825500<br>H 0.62516800 1.62463400 0.03575500<br>H -0.82439700 -2.42345500 -0.10634300<br>H 1.50055300 -3.26066300 -0.10957400<br>H -1.65746100 1.31080000 0.05585400<br>H -2.80797600 -1.52844700 -0.16932400<br>H 2.55930000 2.41171700 1.17154700<br>H 2.52485700 2.73511100 -0.59116600<br>H 4.08033800 2.65815400 0.27335800<br>H 4.16235600 -1.36270100 0.76440900<br>H -5.88583900 -0.01099700 -0.11936400<br>H 2.90277800 -1.30896900 3.01292900<br>O 4.44817500 -1.05153900 2.00426100<br>O 3.25584100 -0.58501000 2.46923400	Zero-point correction= 0.199359 (Hartree/Particle)<br>Thermal correction to Energy= 0.215362<br>Thermal correction to Enthalpy= 0.216306<br>Thermal correction to Gibbs Free Energy= 0.153792<br>Sum of electronic and zero-point Energies= -838.602402<br>Sum of electronic and thermal Energies= -838.586399<br>Sum of electronic and thermal Enthalpies= -838.585455<br>Sum of electronic and thermal Free Energies= -838.647969
**Name**	**TS-4-O4-H-OOH (water)**
Cartesian Coordinates	Frequency and Energy
O 3.25154500 0.88654700 -0.00785100<br>O 3.64392100 -1.73382200 -0.11014200<br>O -5.04795700 -0.57138400 -0.15579400<br>O -4.15573100 1.46870700 0.08262300<br>C -0.22587300 -0.30673100 -0.01597200<br>C 2.15439400 0.12010000 -0.02061300<br>C 0.84509300 0.59049300 0.01227600<br>C 0.01874100 -1.69763400 -0.06148500<br>C 2.39889000 -1.28475100 -0.06449600<br>C 1.30767100 -2.17333000 -0.07278400<br>C -1.57698500 0.24996600 0.00785600<br>C -2.71999600 -0.44112200 -0.08854200<br>C 3.08228800 2.29366700 0.18169900<br>C -4.00920000 0.27018500 -0.04255000<br>H 0.64147100 1.65302000 0.05349700<br>H -0.80642800 -2.39813900 -0.07354700<br>H 1.51936000 -3.23561200 -0.09729000<br>H -1.63954900 1.33063400 0.10910300<br>H -2.76448600 -1.51679700 -0.20353600	Zero-point correction= 0.198207 (Hartree/Particle)<br>Thermal correction to Energy= 0.214399<br>Thermal correction to Enthalpy= 0.215343<br>Thermal correction to Gibbs Free Energy= 0.151628<br>Sum of electronic and zero-point Energies= -838.608484<br>Sum of electronic and thermal Energies= -838.592292<br>Sum of electronic and thermal Enthalpies= -838.591348<br>Sum of electronic and thermal Free Energies= -838.655064

H 2.55924800 2.48685700 1.12056700	
H 2.53276500 2.72893800 -0.65505300	
H 4.08629800 2.70751800 0.22103000	
H 4.15000100 -1.42671700 0.79220300	
H -5.87351700 -0.06233000 -0.11769400	
H 2.71619900 -1.49496200 2.85395800	
O 4.37247700 -1.09507200 2.07600300	
O 3.13761800 -0.70083700 2.48040500	
**Name**	**TS-4-ANION-O4-H-OOH (water)**
Cartesian Coordinates	Frequency and Energy
O 3.25233900 0.88903500 -0.01088100	Zero-point correction= 0.185377 (Hartree/Particle)
O 3.64631200 -1.73398500 -0.11964500	Thermal correction to Energy= 0.201398
O -5.06300500 -0.53685400 -0.28095200	Thermal correction to Enthalpy= 0.202342
O -4.14993400 1.46249800 0.13352700	Thermal correction to Gibbs Free Energy= 0.138838
C -0.23073900 -0.30203800 0.01536200	Sum of electronic and zero-point Energies= -838.165762
C 2.15281400 0.12095800 -0.01123400	Sum of electronic and thermal Energies= -838.149741
C 0.84470300 0.59183200 0.03287200	Sum of electronic and thermal Enthalpies= -838.148797
C 0.01701400 -1.69211900 -0.02537500	Sum of electronic and thermal Free Energies= -838.212300
C 2.39726000 -1.28180600 -0.05505900	
C 1.30697800 -2.16851400 -0.04803100	
C -1.58640500 0.25402600 0.04161500	
C -2.72418400 -0.43389200 -0.10247200	
C 3.08179400 2.29785300 0.15797900	
C -4.07932700 0.22484100 -0.07801900	
H 0.64298100 1.65479200 0.07303300	
H -0.80846100 -2.39255400 -0.02403500	
H 1.51724400 -3.23132100 -0.06994400	
H -1.65035000 1.33076600 0.17678900	
H -2.72987600 -1.50723900 -0.26047300	
H 2.56751000 2.50630800 1.09851300	
H 2.52298500 2.71921300 -0.67993300	
H 4.08505900 2.71505500 0.18094800	
H 4.15958500 -1.43577700 0.76471400	
H 2.73948700 -1.51670000 2.84778700	
O 4.39688300 -1.10656300 2.07821100	
O 3.16311100 -0.71809300 2.48627000	
**Name**	**TS-5-O4-H-OOH (pentyl ethanoate)**
Cartesian Coordinates	Frequency and Energy
O -0.79355100 2.03279300 -0.82802700	Zero-point correction= 0.232035 (Hartree/Particle)
O -2.42639000 -2.38018400 -0.15486700	Thermal correction to Energy= 0.250565
O -2.83719000 0.33327000 -0.78911400	Thermal correction to Enthalpy= 0.251509
O 4.98536400 0.23527300 0.92253600	Thermal correction to Gibbs Free Energy= 0.183097
O 2.84338100 0.56026900 1.46206000	Sum of electronic and zero-point Energies= -953.071619
C 0.97805300 -1.16204200 -0.42919600	Sum of electronic and thermal Energies= -953.053089
C -0.48728500 0.72530000 -0.67605100	Sum of electronic and thermal Enthalpies= -953.052144
C -1.41523000 -1.50612000 -0.30036400	Sum of electronic and thermal Free Energies= -953.120556
C -1.63063700 -0.12664200 -0.55836400	
C 0.79371700 0.21561400 -0.62702500	
C -0.12136200 -2.00993300 -0.28854200	
C 2.31380100 -1.77655000 -0.40567300	
C 3.47295200 -1.31327000 0.08016000	
C 0.28085500 2.94401800 -1.05229300	
C -3.59209100 -1.97597300 0.57706600	
C 3.67794800 -0.08155100 0.87666700	

H 1.65146000 0.86009700 -0.74713400	
H 0.01688100 -3.07429200 -0.13820800	
H 2.35183600 -2.76418800 -0.85981000	
H 4.36673300 -1.90982200 -0.06035200	
H -3.10886400 1.12051100 -0.07323200	
H 0.82320500 2.68039500 -1.96320500	
H 0.96435100 2.95777000 -0.19906600	
H -0.18072500 3.92168600 -1.16999900	
H -3.98408300 -2.88635300 1.02870200	
H -4.33518300 -1.53742500 -0.08627800	
H -3.32652200 -1.26367200 1.36102400	
H 5.08136700 1.01974100 1.48386400	
H -1.44516400 2.10969700 1.22021600	
O -3.26449500 1.81371300 1.00315900	
O -2.11684200 1.58244700 1.68869800	
**Name**	**TS-5-O4-H-OOH (water)**
Cartesian Coordinates	Frequency and Energy
O -0.76894900 2.01608200 -1.03707800	Zero-point correction= 0.230734 (Hartree/Particle)
O -2.47806200 -2.33378600 -0.20013900	Thermal correction to Energy= 0.249479
O -2.83523400 0.38263500 -0.85619700	Thermal correction to Enthalpy= 0.250423
O 4.78144300 0.41555000 0.90028200	Thermal correction to Gibbs Free Energy= 0.181305
O 2.65146900 0.44906000 1.58080900	Sum of electronic and zero-point Energies= -953.078087
C 0.96054200 -1.17465000 -0.48952300	Sum of electronic and thermal Energies= -953.059342
C -0.47748400 0.72049800 -0.81707200	Sum of electronic and thermal Enthalpies= -953.058398
C -1.43177700 -1.48622200 -0.36888600	Sum of electronic and thermal Free Energies= -953.127516
C -1.62495700 -0.11450400 -0.65964300	
C 0.79928000 0.19426200 -0.73774100	
C -0.15063600 -2.00974700 -0.33064000	
C 2.29574500 -1.79067200 -0.42388900	
C 3.41696800 -1.30315500 0.11882200	
C 0.31933900 2.94304700 -1.05138000	
C -3.56468000 -1.90132400 0.63934500	
C 3.52966900 -0.06712400 0.92570100	
H 1.66698500 0.82229300 -0.88393000	
H -0.02221600 -3.06958100 -0.14461200	
H 2.36514900 -2.77016800 -0.89022700	
H 4.34035000 -1.86058500 0.01343700	
H -3.06194700 1.08381700 -0.07069900	
H 0.98021900 2.74438300 -1.89717800	
H 0.87827900 2.88434100 -0.11423800	
H -0.13211500 3.92600200 -1.15714700	
H -3.92798900 -2.79684900 1.14080700	
H -4.36038200 -1.46199400 0.04059700	
H -3.21045200 -1.18265000 1.38014800	
H 4.82544000 1.20285100 1.46670200	
H -1.23175600 1.89617800 1.42537200	
O -3.06684500 1.72070000 1.11037200	
O -1.92910400 1.26588600 1.68517900	
**Name**	**TS-5-ANION-O4-H-OOH (water)**
Cartesian Coordinates	Frequency and Energy
O -0.70014200 2.01101100 -0.98137100	Zero-point correction= 0.217868 (Hartree/Particle)
O -2.51687100 -2.31946800 -0.28651000	Thermal correction to Energy= 0.236444
O -2.80771200 0.41328400 -0.88954600	Thermal correction to Enthalpy= 0.237388
O 4.30632300 0.87575200 0.08285400	Thermal correction to Gibbs Free Energy= 0.168403

O	2.89549000	0.40066000	1.75398100	Sum of electronic and zero-point Energies= -952.637951<br>Sum of electronic and thermal Energies= -952.619375<br>Sum of electronic and thermal Enthalpies= -952.618430<br>Sum of electronic and thermal Free Energies= -952.687416
C	0.95932000	-1.22588700	-0.44845400	
C	-0.44212600	0.70380700	-0.77848500	
C	-1.44547200	-1.48999200	-0.40784200	
C	-1.60710400	-0.11060200	-0.67155400	
C	0.82231300	0.15125000	-0.67635400	
C	-0.17711600	-2.03774800	-0.33586400	
C	2.28062800	-1.85805000	-0.31733800	
C	3.39254100	-1.28996500	0.16107600	
C	0.40914800	2.91203100	-0.95223000	
C	-3.59238200	-1.89290400	0.56937300	
C	3.52912700	0.11212800	0.71041100	
H	1.69947000	0.77056900	-0.80332300	
H	-0.07568900	-3.10191800	-0.15706700	
H	2.32640000	-2.89846300	-0.62749500	
H	4.31166600	-1.86963600	0.17090500	
H	-3.04253400	1.09033300	-0.10335000	
H	1.08507600	2.71510200	-1.78657300	
H	0.94582400	2.82274100	-0.00446700	
H	-0.01665200	3.90744100	-1.04842100	
H	-3.98728100	-2.79788800	1.02844400	
H	-4.37175900	-1.39995000	-0.00904700	
H	-3.21869700	-1.22132100	1.34432200	
H	-1.23573300	1.84367100	1.47082100	
O	-3.06708500	1.70641300	1.12121600	
O	-1.94812200	1.21932200	1.70254100	
**Name**				**TS-6-O4-H-OOH (pentyl ethanoate)**
Cartesian Coordinates				Frequency and Energy
O	-2.45639700	1.02363900	-0.08779900	Zero-point correction= 0.193997 (Hartree/Particle)<br>Thermal correction to Energy= 0.209203<br>Thermal correction to Enthalpy= 0.210147<br>Thermal correction to Gibbs Free Energy= 0.148360<br>Sum of electronic and zero-point Energies= -800.522216<br>Sum of electronic and thermal Energies= -800.507010<br>Sum of electronic and thermal Enthalpies= -800.506066<br>Sum of electronic and thermal Free Energies= -800.567853
O	-3.06307400	-1.50566800	-0.59553200	
O	3.36036000	-0.73446000	-0.99561400	
O	3.11147700	1.42894000	-0.49650200	
C	0.76821000	-0.58183400	0.59767500	
C	2.18463300	-0.22785800	0.99417200	
C	-0.17856600	0.43304500	0.48067900	
C	-1.47698200	0.13433400	0.07168700	
C	0.42064200	-1.91200000	0.31248400	
C	-1.84023300	-1.22028600	-0.20838200	
C	-0.86726000	-2.22625400	-0.06918300	
C	2.93315900	0.27272300	-0.22044400	
C	-2.23310400	2.35529000	0.36245900	
H	2.69515100	-1.10423800	1.39442000	
H	2.18852800	0.57025400	1.73563300	
H	0.10646300	1.45390800	0.70321700	
H	1.16848200	-2.69141500	0.40283100	
H	-1.16235000	-3.24705800	-0.28256100	
H	-1.44642600	2.83863700	-0.22230000	
H	-1.97120500	2.36130000	1.42364200	
H	-3.17338500	2.88059900	0.21306200	
H	-3.76155300	-1.16693100	0.17335200	
H	3.78470900	-0.35437300	-1.78060800	
H	-3.03799500	-1.54682700	2.59738800	
O	-4.30350100	-0.97459500	1.35407000	
O	-3.21574200	-0.71267200	2.13178800	

**Name**				**TS-6-O4-H-OOH (water)**	
Cartesian Coordinates				Frequency and Energy	
O	-2.46783100	1.02563900	-0.09609700	Zero-point correction=	0.193036 (Hartree/Particle)
O	-3.08212100	-1.50333700	-0.59021700	Thermal correction to Energy=	0.208333
O	2.85327300	-0.48091600	-1.23808700	Thermal correction to Enthalpy=	0.209277
O	3.73596700	1.22077600	-0.08988100	Thermal correction to Gibbs Free Energy=	0.147107
C	0.75310400	-0.58116400	0.61584600	Sum of electronic and zero-point Energies=	-800.529206
C	2.16257700	-0.22957600	1.01866800	Sum of electronic and thermal Energies=	-800.513909
C	-0.18621000	0.43728700	0.47827000	Sum of electronic and thermal Enthalpies=	-800.512965
C	-1.48368700	0.13375300	0.07185500	Sum of electronic and thermal Free Energies=	-800.575134
C	0.39846700	-1.91625900	0.35868000		
C	-1.84722800	-1.22171300	-0.18879000		
C	-0.88736600	-2.23296800	-0.02567200		
C	2.99428000	0.26697400	-0.13918900		
C	-2.20916100	2.38385800	0.26753600		
H	2.67440600	-1.11379000	1.40967000		
H	2.17461400	0.54631300	1.78302300		
H	0.10258100	1.46006900	0.68640900		
H	1.14270500	-2.69572900	0.47316100		
H	-1.18715100	-3.25622800	-0.21874300		
H	-1.41836500	2.80274300	-0.35751600		
H	-1.93210100	2.44585700	1.32191000		
H	-3.14021000	2.91692000	0.09434000		
H	-3.76191200	-1.23041900	0.19857600		
H	3.42681700	-0.12988800	-1.93865000		
H	-2.89225300	-1.68843500	2.50838800		
O	-4.26159200	-1.00114000	1.43108900		
O	-3.12756300	-0.81242700	2.15505200		

**Name**				**TS-6-ANION-O4-H-OOH (water)**	
Cartesian Coordinates				Frequency and Energy	
O	-2.50520700	1.00329500	-0.10407000	Zero-point correction=	0.180110 (Hartree/Particle)
O	-3.07499900	-1.54244000	-0.58312900	Thermal correction to Energy=	0.195140
O	2.58125200	0.18194400	-1.34940300	Thermal correction to Enthalpy=	0.196084
O	4.27484000	0.38481300	0.09236800	Thermal correction to Gibbs Free Energy=	0.134955
C	0.75107600	-0.53853900	0.62457700	Sum of electronic and zero-point Energies=	-800.088371
C	2.15456700	-0.17439100	1.00165600	Sum of electronic and thermal Energies=	-800.073342
C	-0.21263900	0.45760600	0.47804100	Sum of electronic and thermal Enthalpies=	-800.072398
C	-1.50369100	0.12826000	0.07372800	Sum of electronic and thermal Free Energies=	-800.133526
C	0.41379800	-1.88159700	0.37523700		
C	-1.84345000	-1.23359900	-0.17638200		
C	-0.86600600	-2.22554100	-0.00671600		
C	3.07213400	0.15483500	-0.19616900		
C	-2.26610100	2.36896300	0.24087900		
H	2.62845200	-0.99023200	1.55194900		
H	2.16333200	0.70549700	1.65013100		
H	0.05641900	1.48781800	0.67535800		
H	1.17129700	-2.64832700	0.49289800		
H	-1.14701200	-3.25539700	-0.19383400		
H	-1.48150000	2.79156600	-0.38958700		
H	-1.98952100	2.45048900	1.29415600		
H	-3.20453400	2.88700200	0.06112300		
H	-3.75816500	-1.27791600	0.18726900		
H	-2.88375900	-1.68675300	2.52249100		
O	-4.27656100	-1.04937300	1.44451400		

O -3.14680100 -0.82189600 2.16119400	
**Name**	**TS-7-O4-H-OOH (pentyl ethanoate)**
Cartesian Coordinates	Frequency and Energy
O -3.03757300 0.99321500 1.17065700<br>O 3.32399700 -1.90535400 -0.23479100<br>O 2.90422700 1.50573900 -0.05488500<br>O 3.67283800 0.18505700 1.59015500<br>C 0.41340000 -0.17546200 -0.74978400<br>C 1.69434300 -0.60105400 -1.43042000<br>C -0.18333500 -1.01927700 0.19542900<br>C -0.18283600 1.06011300 -1.04503200<br>C -1.34903600 -0.64554600 0.83654600<br>C -1.35464900 1.44209000 -0.42575100<br>C -1.95177900 0.60064000 0.54147900<br>C 2.77734400 -0.85317700 -0.40522800<br>C 3.17490000 0.32906100 0.51431800<br>H 1.55247400 -1.52893700 -1.98801600<br>H 2.03206300 0.17980200 -2.11558900<br>H 0.27084900 -1.97813900 0.42469300<br>H 0.28697900 1.71566000 -1.76974800<br>H -1.81817400 -1.28922200 1.57141300<br>H -1.82841100 2.39185400 -0.64547400<br>H -3.90117700 0.31282600 0.91442100<br>H 3.16311500 2.21697700 0.55395900<br>H -4.01114100 -0.79637300 -1.36235300<br>O -4.70948900 -0.41995700 0.32367400<br>O -3.91057100 -1.18102200 -0.47524800	Zero-point correction= 0.169616 (Hartree/Particle)<br>Thermal correction to Energy= 0.184341<br>Thermal correction to Enthalpy= 0.185285<br>Thermal correction to Gibbs Free Energy= 0.123714<br>Sum of electronic and zero-point Energies= -799.338398<br>Sum of electronic and thermal Energies= -799.323672<br>Sum of electronic and thermal Enthalpies= -799.322728<br>Sum of electronic and thermal Free Energies= -799.384300
**Name**	**TS-7-O4-H-OOH (water)**
Cartesian Coordinates	Frequency and Energy
O -3.08014600 0.65804700 1.40826200<br>O 3.33837000 -1.81827800 -0.08593600<br>O 2.76734700 1.59447700 -0.20092700<br>O 4.03916800 0.45736300 1.26082700<br>C 0.37424200 -0.31152900 -0.62444500<br>C 1.64594900 -0.68060200 -1.35461700<br>C -0.12789800 -1.17016500 0.36666500<br>C -0.30869800 0.87258700 -0.92704500<br>C -1.29191200 -0.86169700 1.03947300<br>C -1.47470300 1.19694300 -0.26110000<br>C -1.97912800 0.33580500 0.74019100<br>C 2.78745500 -0.79014200 -0.37824300<br>C 3.27468200 0.49676700 0.33460400<br>H 1.54038100 -1.65134700 -1.84171100<br>H 1.88526300 0.07959500 -2.10023700<br>H 0.40317500 -2.08836800 0.59622000<br>H 0.08538000 1.53401900 -1.69011900<br>H -1.69548200 -1.51648900 1.80277800<br>H -2.01867600 2.10788600 -0.48437000<br>H -3.97046300 0.37315500 0.78756700<br>H 3.09859300 2.37799600 0.27092800<br>H -3.67281800 0.06685200 -1.63669500<br>O -4.73213300 -0.08938400 -0.10057100<br>O -3.85959300 -0.63397800 -0.98707600	Zero-point correction= 0.169525 (Hartree/Particle)<br>Thermal correction to Energy= 0.183996<br>Thermal correction to Enthalpy= 0.184940<br>Thermal correction to Gibbs Free Energy= 0.124653<br>Sum of electronic and zero-point Energies= -799.344178<br>Sum of electronic and thermal Energies= -799.329707<br>Sum of electronic and thermal Enthalpies= -799.328763<br>Sum of electronic and thermal Free Energies= -799.389050
**Name**	**TS-7-ANION-O4-H-OOH (water)**

Cartesian Coordinates	Frequency and Energy
O -3.05629900 0.69444900 1.38880300	Zero-point correction= 0.156392 (Hartree/Particle)
O 3.50560000 -1.77128600 -0.31105300	Thermal correction to Energy= 0.170759
O 2.74255500 1.56824100 0.00521900	Thermal correction to Enthalpy= 0.171703
O 3.77605100 0.26421300 1.51513700	Thermal correction to Gibbs Free Energy= 0.111130
C 0.38871700 -0.30847400 -0.65482700	Sum of electronic and zero-point Energies= -798.913026
C 1.66021000 -0.68070200 -1.37332600	Sum of electronic and thermal Energies= -798.898659
C -0.07510000 -1.11616100 0.39717200	Sum of electronic and thermal Enthalpies= -798.897715
C -0.33669800 0.83432500 -1.01535000	Sum of electronic and thermal Free Energies= -798.958288
C -1.23564100 -0.79866000 1.07256400	
C -1.49785600 1.16937300 -0.34698800	
C -1.96041800 0.35908700 0.71518200	
C 2.82503400 -0.77657000 -0.41061800	
C 3.14255300 0.47764100 0.46307700	
H 1.55543900 -1.65076200 -1.86379600	
H 1.89906900 0.07557000 -2.12441100	
H 0.48460600 -2.00407300 0.67399300	
H 0.02508900 1.45979200 -1.82355600	
H -1.60593500 -1.41472400 1.88356400	
H -2.06937500 2.05167500 -0.61329900	
H -3.94672300 0.41870300 0.78194200	
H -3.68118200 0.03225800 -1.63571600	
O -4.73334200 -0.07151600 -0.09013900	
O -3.87659200 -0.65088100 -0.97007800	
**Name**	**TS-8-O3-H-OOH (pentyl ethanoate)**
Cartesian Coordinates	Frequency and Energy
O -4.17715300 -1.51005200 -0.30943900	Zero-point correction= 0.165265 (Hartree/Particle)
O -4.34862500 0.64439900 0.26308600	Thermal correction to Energy= 0.178950
C -0.05392000 1.00103600 -0.04019400	Thermal correction to Enthalpy= 0.179894
C 0.78656800 0.02516000 -0.57347200	Thermal correction to Gibbs Free Energy= 0.121405
C 0.51002800 2.19626200 0.42532900	Sum of electronic and zero-point Energies= -724.116691
C 2.18270900 0.23024800 -0.62029100	Sum of electronic and thermal Energies= -724.103006
C -1.50909800 0.83096500 0.04844400	Sum of electronic and thermal Enthalpies= -724.102061
C 1.89253000 2.40880800 0.38587600	Sum of electronic and thermal Free Energies= -724.160550
C 2.73009900 1.43618000 -0.11734200	
C -2.18994100 -0.29657100 -0.17450600	
C -3.66106100 -0.29818800 -0.04273000	
H -0.14124500 2.96475700 0.82738000	
H -2.07390500 1.71459500 0.33676000	
H 2.30189400 3.34107500 0.75590400	
H 3.80436300 1.57170900 -0.15971500	
H -1.72116400 -1.23430400 -0.44659700	
H -5.13938300 -1.45394100 -0.20756800	
H 0.39806300 -0.90188100 -0.97800300	
O 2.97467100 -0.67952500 -1.14158100	
H 2.92743100 -1.64101800 -0.52941900	
H 2.28652400 -1.58902300 1.92069800	
O 2.86027400 -2.46238000 0.37702800	
O 1.84457400 -1.98936400 1.15264000	
**Name**	**TS-8-O3-H-OOH (water)**
Cartesian Coordinates	Frequency and Energy
O -3.99071600 -1.24944100 0.05950800	Zero-point correction= 0.164901 (Hartree/Particle)
O -3.78106800 0.97830600 -0.00959000	Thermal correction to Energy= 0.178415
C 0.51305100 0.50563300 -0.02183000	Thermal correction to Enthalpy= 0.179359

C 1.17795200 -0.71773000 -0.00637800<br>C 1.26984400 1.68758200 -0.03977500<br>C 2.58748200 -0.75966800 -0.00383500<br>C -0.94988400 0.61719100 -0.01779800<br>C 2.66739400 1.65441700 -0.03343000<br>C 3.33069000 0.44518000 -0.00718800<br>C -1.81964700 -0.39742000 0.00952600<br>C -3.26626200 -0.12091600 0.01690100<br>H 0.75324000 2.64083200 -0.05416700<br>H -1.34153200 1.63127100 -0.03600100<br>H 3.22635700 2.58183700 -0.04412700<br>H 4.41261800 0.38612700 0.00393600<br>H -1.52355700 -1.43890000 0.03035000<br>H -4.93337100 -1.01866000 0.06604100<br>H 0.64286100 -1.66092300 0.00491300<br>O 3.21577400 -1.92635100 -0.00905700<br>H 3.21734800 -2.33525700 1.05196500<br>H 1.53893300 -1.52146700 2.64369800<br>O 3.10104300 -2.48570200 2.27417800<br>O 2.49452400 -1.33568500 2.66817300	Thermal correction to Gibbs Free Energy= 0.121246<br>Sum of electronic and zero-point Energies= -724.120804<br>Sum of electronic and thermal Energies= -724.107290<br>Sum of electronic and thermal Enthalpies= -724.106346<br>Sum of electronic and thermal Free Energies= -724.164459
**Name**	**TS-8-ANION-O3-H-OOH (water)**
Cartesian Coordinates	Frequency and Energy
O -4.05661300 -1.27455500 0.09605100<br>O -3.82755600 0.94882400 0.01242600<br>C 0.45843700 0.49699700 -0.02102200<br>C 1.13391400 -0.72043600 -0.01302200<br>C 1.21410200 1.68047500 -0.03788000<br>C 2.54368500 -0.75610300 -0.01483300<br>C -1.01019600 0.60019200 -0.00913200<br>C 2.61239900 1.65482900 -0.03673000<br>C 3.28383700 0.44992500 -0.01650000<br>C -1.87241000 -0.41795000 0.02280200<br>C -3.36600700 -0.22107200 0.04398500<br>H 0.69499400 2.63262000 -0.04705400<br>H -1.40526700 1.61280800 -0.02408500<br>H 3.16583200 2.58577800 -0.04660500<br>H 4.36598200 0.39565500 -0.00936900<br>H -1.53841000 -1.45041100 0.04073800<br>H 0.60541900 -1.66735900 -0.00336100<br>O 3.17769300 -1.92176600 -0.02709800<br>H 3.17078400 -2.34100600 1.02200900<br>H 1.49910200 -1.52817200 2.62239800<br>O 3.05460800 -2.50361700 2.25277500<br>O 2.45628700 -1.35263100 2.65677300	Zero-point correction= 0.151892 (Hartree/Particle)<br>Thermal correction to Energy= 0.165247<br>Thermal correction to Enthalpy= 0.166191<br>Thermal correction to Gibbs Free Energy= 0.108129<br>Sum of electronic and zero-point Energies= -723.677871<br>Sum of electronic and thermal Energies= -723.664516<br>Sum of electronic and thermal Enthalpies= -723.663572<br>Sum of electronic and thermal Free Energies= -723.721634

References

1. M. G. Evans and M. Polanyi, *Trans. Faraday Soc.*, 1935, **31**, 875-894.
2. H. Eyring, *J. Chem. Phys.*, 1935, **3**, 107-115.
3. D. G. Truhlar, W. L. Hase and J. T. Hynes, *J. Phys. Chem.*, 1983, **87**, 2664-2682.
4. T. Furuncuoglu, I. Ugur, I. Degirmenci and V. Aviyente, *Macromolecules*, 2010, **43**, 1823-1835.
5. E. Vélez, J. Quijano, R. Notario, E. Pabón, J. Murillo, J. Leal, E. Zapata and G. Alarcón, *J. Phys. Org. Chem.*, 2009, **22**, 971-977.
6. E. Pollak and P. Pechukas, *J. Am. Chem. Soc.*, 1978, **100**, 2984-2991.
7. A. Fernández-Ramos, B. A. Ellingson, R. Meana-Pañeda, J. M. Marques and D. G. Truhlar, *Theor. Chem. Acc.*, 2007, **118**, 813-826.
8. C. Eckart, *Phy. Rev.*, 1930, **35**, 1303.
9. R. A. Marcus, *Annu. Rev. Phys. Chem.*, 1964, **15**, 155-196.
10. R. A. Marcus, *Rev. Mod. Phys.*, 1993, **65**, 599.
11. Y. Lu, A. Wang, P. Shi and H. Zhang, *PloS one*, 2017, **12**, e0169773.
12. Y. Lu, A. Wang, P. Shi, H. Zhang and Z. Li, *PloS one*, 2015, **10**, e0133259.
13. S. F. Nelsen, S. C. Blackstock and Y. Kim, *J. Am. Chem. Soc.*, 1987, **109**, 677-682.
14. S. F. Nelsen, M. N. Weaver, Y. Luo, J. R. Pladziewicz, L. K. Ausman, T. L. Jentzsch and J. J. O'Konek, *J. Phys. Chem. A*, 2006, **110**, 11665-11676.
15. A. Galano and J. R. Alvarez-Idaboy, *J. Comput. Chem.*, 2013, **34**, 2430-2445.
16. F. C. Collins and G. E. Kimball, *J. Colloid Sci.* , 1949, **4**, 425-437.
17. M. Von Smoluchowski, *Z. Phys. Chem*, 1917, **92**, 129-168.
18. D. G. Truhlar, *J. Chem. Educ.*, 1985, **62**, 104.
19. A. Einstein, *Ann. Phys.*, 1905, **17**, 549-560.
20. G. G. Stokes, *Mathematical and Physical Papers*, University Press, Cambridge, 1905.
21. A. V. Marenich, C. J. Cramer and D. G. Truhlar, *The Journal of Physical Chemistry B*, 2009, **113**, 6378-6396.
22. C. P. Kelly, C. J. Cramer and D. G. Truhlar, *Journal of chemical theory and computation*, 2005, **1**, 1133-1152.
23. A. Galano and J. Raúl Alvarez-Idaboy, *Int. J. Quantum Chem.*, 2019, **119**, e25665.

24. Q. V. Vo, T. V. Gon, M. V. Bay and A. Mechler, *J. Phys. Chem. B*, 2019, **123**, 10672-10679.
25. Q. V. Vo and A. Mechler, *J. Chem. Inf. Model.*, 2020, **60**, 316-321.
26. Y. Okuno, *Chem.: Eur. J.*, 1997, **3**, 212-218.
27. S. Benson, *The foundations of chemical kinetics:* , Malabar, Florida, 1982.
28. C. Iuga, J. R. Alvarez-Idaboy and A. Vivier-Bunge, *J. Phys. Chem. B*, 2011, **115**, 12234-12246.
29. J. R. Alvarez-Idaboy, L. Reyes and N. Mora-Diez, *Org. Biomol. Chem.*, 2007, **5**, 3682-3689.
30. T. H. Le, T. T. Tran and L. K. Huynh, *Chemom. Intell. Lab. Syst.*, 2018, **172**, 10-16.
